# Chemoradiotherapy‐induced increase in Th17 cell frequency in cervical cancer patients is associated with therapy resistance and early relapse

**DOI:** 10.1002/1878-0261.13095

**Published:** 2021-09-13

**Authors:** Laura Theobald, Russalina Stroeder, Patrick Melchior, Ioan Iulian Iordache, Tanja Tänzer, Meike Port, Birgit Glombitza, Stefanie Marx, David Schub, Christian Herr, Martin Hart, Nicole Ludwig, Eckart Meese, Yoo‐Jin Kim, Rainer Maria Bohle, Sigrun Smola, Christian Rübe, Erich Franz Solomayer, Barbara Walch‐Rückheim

**Affiliations:** ^1^ Institute of Virology and Center of Human and Molecular Biology Saarland University Homburg/Saar Germany; ^2^ Department of Obstetrics and Gynecology Saarland University Medical Center Homburg/Saar Germany; ^3^ Department of Radiation Oncology Saarland University Medical Center Homburg/Saar Germany; ^4^ Department of Transplant and Infection Immunology Saarland University Homburg/Saar Germany; ^5^ Department of Internal Medicine V ‐ Pulmonology, Allergology and Critical Care Medicine Saarland University Medical Center Homburg/Saar Germany; ^6^ Institute of Human Genetics Saarland University Homburg/Saar Germany; ^7^ Institute of Human Genetics and Center of Human and Molecular Biology Saarland University Homburg/Saar Germany; ^8^ Institute of Pathology Saarland University Medical Center Homburg/Saar Germany

**Keywords:** AKT, cervical cancer recurrence, chemoradiotherapy, resistance, T‐helper‐17 cells

## Abstract

Cervical cancer therapy is still a major clinical challenge, as patients substantially differ in their response to standard treatments, including chemoradiotherapy (CRT). During cervical carcinogenesis, T‐helper (Th)‐17 cells accumulate in the peripheral blood and tumor tissues of cancer patients and are associated with poor prognosis. In this prospective study, we find increased Th17 frequencies in the blood of patients after chemoradiotherapy and a post‐therapeutic ratio of Th17/CD4^+^ T cells > 8% was associated with early recurrence. Furthermore, Th17 cells promote resistance of cervical cancer cells toward CRT, which was dependent on the AKT signaling pathway. Consistently, patients with high Th17 frequencies in pretherapeutic biopsies exhibit lower response to primary CRT. This work reveals a key role of Th17 cells in CRT resistance and elevated Th17 frequencies in the blood after CRT correspond with early recurrence. Our results may help to explain individual treatment responses of cervical cancer patients and suggest evaluation of Th17 cells as a novel predictive biomarker for chemoradiotherapy responses and as a potential target for immunotherapy in cervical cancer.

## Graphical abstract

This study demonstrates that chemoradiotherapy‐treated patients with cervical cancer have increased frequencies of Th17 cells. Th17 frequency was associated with early cancer relapse. Additionally, high pretherapeutic Th17 amounts *in situ* were correlated to a failed response to chemoradiotherapy. Our work suggests that the evaluation of Th17 cells could serve as a novel predictive biomarker for chemoradiotherapy responses and a potential target for immunotherapy in patients with cervical cancers.

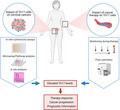

**Abbreviations**
(C/EBPβ): CAAT/enhancer‐binding protein β
aCRT: adjuvant chemoradiotherapy
EGFR: epidermal growth factor receptor
FIGO: International Federation of Gynecology and Obstetrics
HPV: human papillomavirus
IL: interleukin
MAPK: mitogen‐activated protein kinase
MDSCs: myeloid‐derived suppressor cells
OSM: oncostatin M
pCRT: primary chemoradiotherapy
PMA: phorbol‐12‐myristate‐13‐acetate
qRT-PCR: quantitative real-time polymerase chain reaction
STAT3: signal transducer and activator of transcription
Th1: T-helper(Th)-1 cells
Th17: T-helper(Th)-17 cells
Tregs: regulatory T cells
VEGF: vascular endothelial growth factor

## 1 Introduction

Cervical cancer is a consequence of persistent infection with high‐risk human papillomaviruses (HPV) and the fourth most frequent cancer in women worldwide. Standard of care therapy for patients with cervical cancers is a stage‐dependent therapy according to the International Federation of Gynecology and Obstetrics (FIGO) classification, which includes besides surgery alone in early stages, adjuvant platinum‐based concurrent chemoradiotherapy (aCRT) in addition to surgery for advanced cervical cancers with prognostic risk factors for local relapse and primary chemoradiotherapy (pCRT) for locally advanced often inoperable cervical carcinoma (FIGO > IIB). Platinum‐based chemotherapy is applied to patients with advanced, metastatic, or recurrent cervical cancer [1]. However, therapy resistance in a subset of patients is still a major clinical challenge. Depending on initial tumor stage, 8 to 26% of women with cervical cancer experience relapse of disease, most commonly within the first 2 years of completing primary treatment [2]. CRT is applied to kill tumor cells; however, immune cells are also affected during CRT treatment [3]. Different T‐cell subsets react in a differential way to therapeutic approaches showing enhanced sensitivity or survival [4, 5, 6, 7]. This may result in a therapy‐induced micromilieu in the patients` blood and tumor tissues, which may affect progression and clinical outcome of the disease.

Invasive cervical cancers are often associated with strong inflammatory infiltrates in the stroma [8, 9, 10, 11, 12, 13, 14]. Different studies have shown that HPV‐transformed keratinocytes actively contribute to the inflammatory microenvironment during cervical carcinogenesis via production of the cytokine interleukin (IL)‐6 [11, 12, 13, 15]. During cervical carcinogenesis T‐helper(Th)‐17 cells, an IL‐17‐expressing subgroup of Th cells [16] with proinflammatory as well as tumor‐promoting properties in different cancer types [17, 18] increase in the blood of cervical cancer patients [19, 20], infiltrate in cervical cancer tissues [13, 21], and were correlated with poor prognosis for the patients [14, 21]. We previously demonstrated that cervical cancer cells actively contribute to the recruitment of Th17 cells [13]. Although HPV‐infected keratinocytes express only low amounts of the Th17‐attracting chemokine CCL20 as a consequence of the HPV infection [22], they instruct cervical fibroblasts to produce CCL20 in an IL‐6/CAAT/enhancer‐binding protein β (C/EBPβ)‐dependent manner and thereby support Th17 cell recruitment [13]. Furthermore, we recently identified that HPV‐transformed cells also interact with stromal fibroblasts to support Th17 expansion [14]. Using again the IL‐6/C/EBPβ‐pathway, cervical cancer cells induced IL‐1β expression in fibroblasts, which mediated enhanced production of the cytokine IL‐23 in dendritic cells supporting Th17 expansion. The presence of Th17 cells *in situ* correlated with IL‐23‐expressing CD83^+^ cells, advanced tumor stage, lymph node metastases, and retrospectively with cervical cancer recurrence [13, 14].

In this study, we investigated the role of Th17 cells in cervical cancer therapy. We considered both, the impact of different therapeutic approaches on Th17 frequencies in patients` blood and the influence of Th17 cells on the responsiveness of cervical cancer cells toward treatment with chemotherapeutic drug and irradiation. We demonstrate that CRT increased Th17 frequencies in the blood of cervical cancer patients. Notably, post‐therapeutic enhanced Th17 frequencies were associated with recurrent cervical cancers in follow‐up studies. Our data provide evidence that Th17‐instructed cervical cancer cells showed reduced responsiveness toward chemotherapeutic drug, irradiation, and combined treatment and identified Th17‐induced activation of the AKT signaling pathway as the responsible resistance mechanism. Notably, our data show that high pretherapeutic Th17 frequencies and phosphorylated AKT expression correlate with the reduced response to pCRT in cervical cancer patients *in vivo*.

## 2 Methods

### 2.1 Study participants and study design

Peripheral blood samples obtained from 70 cervical cancer patients, treated from 2017 to 2020 in the Saarland University Hospital, and 70 healthy female age‐matched controls (HC) were used for monitoring of Th17 cells. 35 patients were treated with surgery alone, and 35 patients received chemoradiotherapy (*n* = 20 adjuvant chemoradiotherapy (aCRT) and *n* = 15 primary chemoradiotherapy (pCRT)). Blood samples were obtained at primary diagnosis, one day after primary surgery, before the onset of platinum‐based CRT, and directly after completion of CRT. The applied irradiation in CRT consisted of external beam irradiation (EBRT) (*n* = 28) or combined with intracavitary HDR brachytherapy (*n* = 7) and ranged from 45 to 59.92 Gray (Gy) for EBRT and 15–28 Gy for local brachytherapy. Concurrent chemotherapy was platinum‐based, consisted of cisplatin or carboplatin and was applied once weekly with a maximum total number of 6 cycles. Clinicopathologic and demographic data for the patient cohorts are listed in Table 1. Absolute numbers of CD4^+^ T cells/µl were determined using blood counts. Absolute numbers of Th17 cells/µL were calculated by incorporating Th17 frequency values per absolute number of CD4^+^ T cells.

**Table 1:** Clinicopathologic characteristics of patients.

Participants (*n*)	Healthy controls (*n* = 70)	Treated with surgery (*n* = 35)	Treated with CCRT (*n* = 35)	
			aCRT (*n* = 20)	pCRT (*n* = 15)
Age of diagnosis/participation (mean ± SD)	53.1 ± 9.9	53.3 ± 12.5	55.0 ± 13.6	59.1 ± 8.3
Diagnosis:	/			
Squamous cell carcinomas (*n* = 55)		*n* = 27	*n* = 14	*n* = 14
Adenocarcinomas (*n* = 15)		*n* = 8	*n* = 6	*n* = 1
Stage (FIGO)	/			
IA		*n* = 3		
IA1		*n* = 4		
IA2		*n* = 2		
IB		*n* = 3	*n* = 3	
IB1		*n* = 17	*n* = 2	*n* = 2
IB2		*n* = 2		
II				*n* = 1
IIA				*n* = 1
IIA1		*n* = 1		
IIA2		*n* = 3	*n* = 2	
IIB			*n* = 9	*n* = 2
III				*n* = 2
IIIA				*n* = 1
IIIB			*n* = 4	
IIIC				*n* = 2
IV				*n* = 1
IVA				*n* = 2
IVB				*n* = 1
Nodal stage	/			
N0		*n* = 34	*n* = 3	*n* = 6
N1		*n* = 0	*n* = 17	*n* = 9
NX		*n* = 1	*n* = 0	*n* = 0

### 2.2 Ethics statement

This study has been conducted according to Declaration of Helsinki principles. IHC and immunofluorescence stainings of anonymized tissue samples and the usage of peripheral blood mononuclear cells (PBMC) of cervical cancer patients and healthy controls were approved by the Ethics Committees of the Medical Faculty of the Saarland University at the Saarland Ärztekammer (Saarbrücken, Germany). Written informed consent was provided by all study participants.

### 2.3 Tissue specimens, IHC, and immunofluorescence analysis

Formalin‐fixed paraffin‐embedded anonymized lesions of the cervix uteri from 70 patients were taken from the local pathology archive of the Saarland University Medical Center (Homburg, Germany). These included a subset of 20 pretreatment SCC biopsies from patients that had been subjected to pCRT. These tumors were pre‐ and post‐therapeutically staged according to FIGO or TNM categories (Table S1) by expert pathologists (Y.‐J. Kim or R.M. Bohle). Lesions were stained for pThr308‐ or pSer473‐AKT expression and costained for CD4 and IL‐17. Two‐micrometer thick sections fixed on microslides were deparaffinized with xylene and hydrated using a diluted alcohol series. For immunohistochemistry, sections were incubated with citrate buffer (10 mm, pH 7.0), for immunofluorescence with TE buffer (10 mm Tris, 1 mm EDTA, pH 9.0) and cooked for 10 min or 20 min, respectively, for antigen retrieval and immersed in 3% H_2_O_2_ in TBS (50 mm Tris/HCl, 150 mm NaCl, pH 7.6) to block endogenous peroxidase activity. To reduce nonspecific staining, each section was treated with 2.5% normal horse serum for 30 min. Rabbit anti‐pThr308‐AKT antibody (1 : 250, Abcam; AB_722678), rabbit anti‐pSer473‐AKT antibody (1 : 100, Cell Signaling; 2315049), mouse anti‐CD4 monoclonal antibody 4B12 (1 : 1000, Leica Biosystems; AB_563560), and rabbit anti‐IL‐17 polyclonal antibody (1 : 500, Abcam; AB_1603584) were used. For immunohistochemistry, ImmPRESS Detection Kit (Vector Laboratories, Burlingame, USA) was used. For immunofluorescence TSA™ Kit #2, with HRP‐Goat Anti‐Mouse IgG and Alexa Fluor™ 488 Tyramide and TSA™ Kit #13, with HRP‐Goat Anti‐Rabbit IgG and Alexa Fluor® 546 Tyramide (Life Technology, Darmstadt, Germany) were used according to the manufacturer`s instructions. Lesions were scanned with standardized settings using Olympus BX51 microscope and VIS (Visiopharm Integrator system, Hørsholm, Denmark), Cell Sens Dimension and Microsoft Image Composite Editor Program. To evaluate the number of infiltrating Th17 cells, the numbers of CD4^+^ and IL‐17^+^ cells were counted and referred to numbers·mm^−2^. pThr308‐ or pSer473‐AKT staining intensity was classified using the immunoreactive score (IRS; Table S2; refs. [23]).

### 2.4 Isolation and staining of peripheral blood mononuclear cells and flow cytometry

PBMCs were isolated from heparinized blood samples by Pancoll (Pan Biotec) density gradient centrifugation. A part of the PBMCs were stimulated with phorbol‐12‐myristate‐13‐acetate (PMA; 5 ng·mL^−1^)/ionomycin (500 ng·mL^−1^) (both from Sigma‐Aldrich, Taufkirchen, Germany) for 6 h. After 2 h, brefeldin A (10 mg·mL^−1^; Sigma) was added. Cells were fixed using BD Bioscience Cytofix/Cytoperm Kit and stained using anti‐CD4‐PE (AB_395752) and anti‐IL‐17‐APC (AB_1603584) (Table S3) or respective isotype control antibodies (BD Biosciences, Heidelberg and Miltenyi Biotec, Bergisch‐Gladbach, Germany) and analyzed by flow cytometry (FACSCantoII; BD Biosciences). For long‐term storage, cells were cryoconserved in 90% fetal calf serum (FCS) and 10% DMSO in liquid nitrogen.

### 2.5 Generation and cultivation of Th17 cells *in vitro* and ELISA

Generation and isolation of Th17 cells *in vitro* was done as previously described [13], [24]. The isolated Th17 cells were used for coculture experiments with cervical cancer cells in cytotoxicity assays as well as for generation of conditioned media (CM). For CM, cells were cultured at a density of 1 × 10^6^/mL in RPMI1640 medium (Sigma, Taufkirchen, Germany) plus supplements (10% heat‐inactivated endotoxin‐tested FCS (Biochrom, Berlin, Germany) and 1 mm sodium pyruvate) and were stimulated using T‐cell Activation/Expansion Kit (Miltenyi Biotech, Bergisch Gladbach, Germany). After 24 h, CM were collected and analyzed for IL‐17A by DuoSet ELISA (R&D Systems, Minneapolis, USA). Concentrations of IL‐17A in the used CM ranged from 827.4 to 964.9 pg·mL^−1^.

### 2.6 Cell culture and cytotoxicity assays

HPV16‐positive cervical carcinoma cell lines SiHa (RRID:CVCL_0032) and CaSki (RRIID:CVCL_1100) and HPV18‐positive cell lines SW756 (RRID:CVCL_1727) and HeLa (RRID:CVCL_0030) were obtained from M. von Knebel‐Doeberitz before 2000 and authenticated regularly by the German collection of microorganisms and cell cultures (DSMZ) using short tandem repeat DNA typing, last in August 2020. Cell lines used in this study were maintained in a 37 °C and 5% CO_2_ incubator. SiHa, CaSki, SW756, and HeLa cells were cultured in DMEM (Sigma‐Aldrich, Taufkirchen, Germany) supplemented with 10% heat‐inactivated endotoxin‐tested FCS (Biochrom), 1 mm sodium pyruvate, and 2 mm l‐alanyl‐l‐glutamine. Mycoplasma testing was performed monthly using MycoTrace PCR detection kit (PAA, Heidelberg, Germany) or Venor®GeM Classic (Minerva Biolabs; Berlin, Germany). For cytotoxicity assays, 5 × 10^3^ (SiHa), 1 × 10^4^ (CaSki), 3 × 10^3^ (HeLa), or 2 × 10^4^ (SW756) cells/96‐well culture dish were stimulated with 100 ng·mL^−1^ IL‐17A (Miltenyi Biotech) or CM of Th17 cells (1 : 20 diluted) for 24 h or medium as a control. Neutralizing anti‐IL‐17 antibody or matched isotype control antibody (1 μg·mL^−1^; R&D Systems) was added to CM 2 h before usage. For chemotherapy experiments, cells were challenged with serial dilutions of the chemotherapeutic drug cisplatin for 18 h. For irradiation experiments, cells were subsequently irradiated with a dose of 6 Gy. In chemoradiotherapy experiments, cells were challenged with a cisplatin concentration of 1.56 µg·mL^−1^ (Hexal, Holzkirchen, Germany) for 2 h as a radio sensitizer and subsequently irradiated with a dose of 6 Gy. Irradiation was performed using a linear accelerator (Oncor™; Siemens AG). Computed‐tomography‐based three‐dimensional dose calculations were made with the Pinnacle™ planning system (Philips Radiation Oncology Systems) previously. To improve photon dose homogeneity, the plates were on top of a 1‐cm‐thick plexiglass leaf. The radiation characteristics were as follows: size of the radiation field 30 × 30 cm; collimator angle 0°; gantry angle 180 °; beam energy 6 MV photons; dose‐rate 2 Gy·min^−1^. Cell viability was assessed 48 h later by the neutral red uptake method as described previously [25].

### 2.7 Protein expression analysis by western blot analysis

Carcinoma cells were seeded at a density of 8 × 10^5^ (SiHa), 5 × 10^5^ (HeLa), or 1 × 10^6^ (SW756) cells/6‐well culture dish. 24 h later, they were incubated with medium or conditioned media of Th17 cells (diluted 1 : 4) for 15 min. To prepare whole cell extracts, cells were lysed with 2× lysis buffer (130 mm Tris/HCl, 6% SDS, 10% 3‐Mercapto‐1,2‐propanediol, 10% glycerol) and 3 times treated with ultrasound for 5 s. 15 µg of the whole protein extracts was separated by SDS gel electrophoresis and transferred to a nitrocellulose membrane (Whatman, GE Healthcare, Freiburg, Germany). Rabbit anti‐pThr308‐AKT (1 : 1000; D25E6; Cell Signaling), rabbit anti‐pSer473‐AKT (1 : 2000; D9E; Cell Signaling), rabbit anti‐AKT1 (1 : 1000; C73H10; Cell Signaling), rabbit anti‐AKT2 (1 : 1000; D6G4; Cell Signaling), rabbit anti‐AKT3 (1 : 1000; E1Z3W; Cell Signaling), rabbit anti‐panAKT (1 : 1000; C67E7; Cell Signaling), and mouse anti‐β‐Actin antibody (1 : 5000; AC‐15; Sigma‐Aldrich) were used. Secondary Abs (Sigma‐Aldrich) and SuperSignal West Dura Substrate (Thermo Fisher Scientific, Schwerte, Germany) were used for detection with ChemiDoc XRS+ Molecular Imager. Expression was quantified with the Image Lab software (both Bio‐Rad, Feldkirchen, Germany).

### 2.8 SiRNA transfections

30 pmol of indicated siRNAs with respective target sequences (ON‐TARGETplus Nontargeting siRNA #2, ON‐TARGETplus siRNA #12 (CAAGGGCACUUUCGGCAAG) and #13 (UCACAGCCCUGAAGUACUC) for human AKT1, siRNA #9 (ACACAAGGUACUUCGAUGA) and #10 (GCAAGGCACGGGCUAAAGU) for human AKT2, siRNA #11 (UCGAGUAGCUAUCAAGAAA) and #14 (ACACCAACCUCUCGUACAU) for MAPK1 and siRNA #20 (GGAAUUCAAUGAUGUGUAU) and #21 (UCUCCGAGGUCUAAAGUAU) for MAPK14, all from Horizon, Cambridge, United Kingdom) per 2.5 × 10^5^ cells/6‐well culture dish were transfected with Lipofectamine RNAiMax (Invitrogen). Efficient knock down was confirmed by western blot analysis. Cells were stimulated as described above and used in cytotoxicity assays.

### 2.9 qRT‐PCR

RNA was isolated using Qiazol (Qiagen, Hilden, Germany), and cDNA was generated from 1 µg of RNA with SuperscriptII (Invitrogen, Carlsbad, CA, USA). Real‐time PCR was performed with the LightCycler1.5 instrument (Roche, Mannheim, Germany). PCR primers (Sigma‐Aldrich) and probes (Roche Universal Probe Library; Roche) were designed using the Probe Finder software version 2.53 (Roche) as previously described [13]. The 75‐bp fragment of AKT3 was detected with primers 5′‐TTGCTTTCAGGGCTCTTGAT‐3′ and 5′‐CATAATTTCTTTTGCATCATCTGG‐3′ and probe no. 22; the 94‐bp fragment of RPL13A was detected with primers 5′‐AGCGGATGAACACCAACC‐3′ and 5′‐TTTGTGGGGCAGCATACTC‐3′ and probe no. 28.

### 2.10 Statistics

All statistical analyses were performed using the graphpad Prism 8 (graphpad Software) program. To evaluate the statistical differences between the analyzed groups, a Mann–Whitney *U*‐test was applied for the comparison of nonparametric data between two groups and the Kruskal–Wallis test for comparison of nonparametric data of > 2 groups. Significances are indicated by asterisks (*P < 0.05; **P < 0.01; ***P < 0.001; ****P < 0.0001). Correlation between IRS of pThr308‐AKT or pSer473‐AKT and the number of Th17 cells was done using Spearman rank correlation. Best cutoffs to discriminate patients with increased Th17 frequencies and with or without recurrent cervical cancers were identified by receiver operator characteristics (ROC) analysis and Youden’s index calculation.

## 3 Results

### 3.1 Increase in Th17 frequencies after chemoradiotherapy in cervical cancer patients

**Fig. 1:**
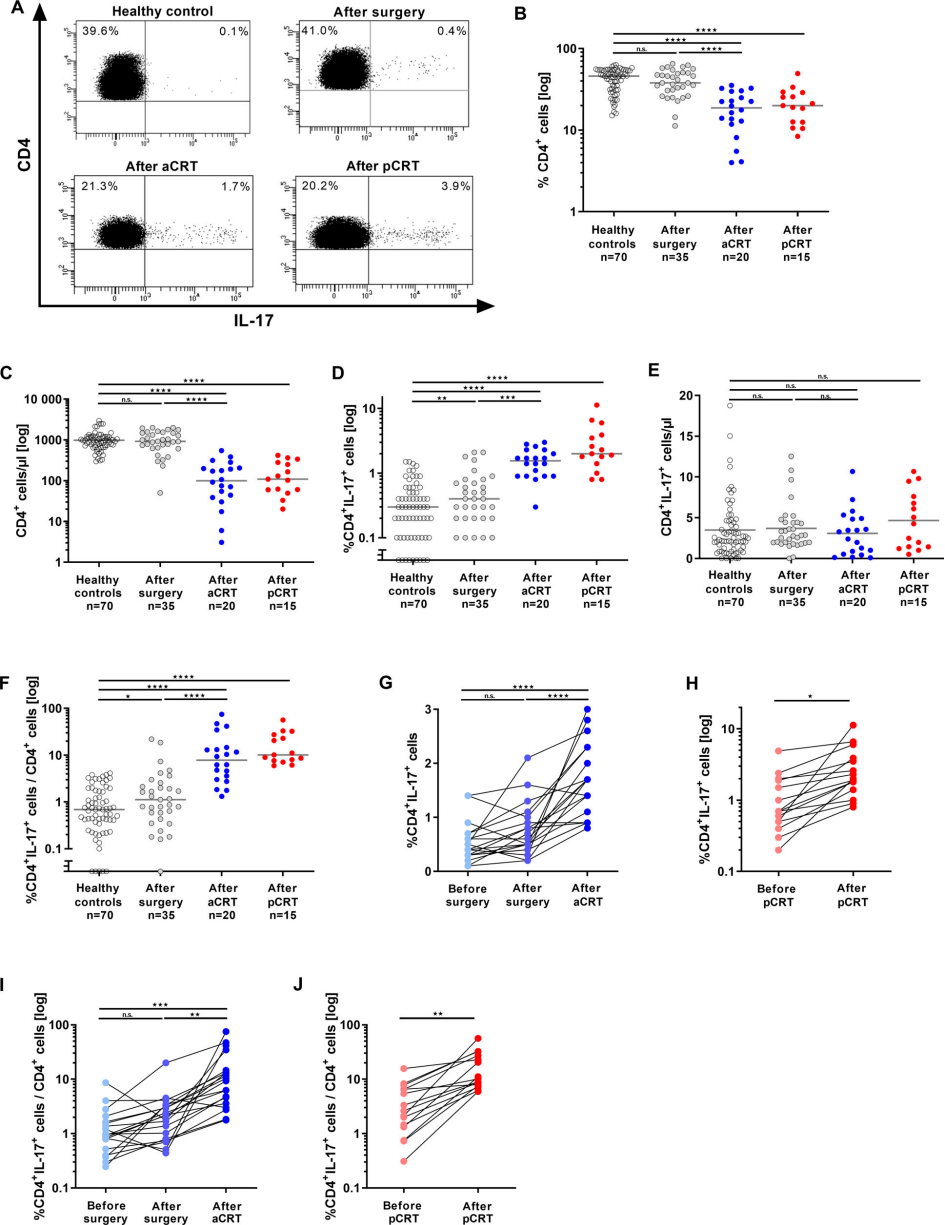
Increased frequencies of Th17 cells after aCRT and pCRT in the blood of cervical cancer patients. PBMCs of 70 cervical cancer patients and 70 female age‐matched healthy controls were analyzed for Th17 frequencies by flow cytometry. (A) Shown is one representative dot blot of the analyzed groups. Numbers indicate percentages of CD4^+^ T cells or CD4^+^IL‐17^+^ cells, respectively. (B) Frequencies of CD4^+^ T cells and (D) CD4^+^IL‐17^+^ cells out of lymphocytes. (C, E) Absolute numbers/µL of CD4^+^ T cells and Th17 cells and (F) proportions of Th17 per total CD4^+^ T cells were determined. Healthy controls (*n* = 70), patients treated with surgery alone (*n* = 35; gray dots), aCRT (adjuvant chemoradiotherapy; *n* = 20; blue dots), and pCRT (primary chemoradiotherapy; *n* = 15; red dots). Gray line: median value of the respective groups. (G, H) PBMCs of cervical cancer patients which received aCRT ((G); *n* = 20) or pCRT ((H); *n* = 15) were analyzed for Th17 frequencies and (I, J) proportions of Th17 per total CD4^+^ T cells during therapy. *P*‐value according to the nonparametric Mann–Whitney *U*‐test (H, J) or Kruskal–Wallis test (B, C, D, E, F, G, I). Asterisks represent statistical significances: n.s.: not significant; *P < 0.05; **P < 0.01; ***P < 0.001; ****P < 0.0001.

We analyzed peripheral blood specimens obtained from 70 cervical cancer patients (Table 1) treated with surgery alone, adjuvant CRT (aCRT) in addition to surgery or solely primary CRT (pCRT) and age‐matched 70 female HC (representative dot plots are shown in Fig. 1A). We found that frequencies and absolute numbers of CD4^+^ T cells were not affected by surgery alone in comparison with HC. However, in the cohort of aCRT and pCRT percentages (Fig. 1B) and absolute numbers of CD4^+^ T cells/µL (Fig. 1C) were significantly decreased. When we investigated Th17 cells, we found that pretherapeutic frequencies of Th17 cells in the patients` blood of the analyzed cohorts correlated with advanced tumors based on FIGO stages (Fig. S1A; *r* = 0.4386, *P* = 0.0005). In contrast to CD4^+^ T cells, frequencies of Th17 cells were significantly increased during cervical cancer therapy (Fig. 1D). In patients with surgery alone, the Th17 frequency (median 0.4%) was significantly higher than in HC (median 0.3%). Notably, adjuvant CRT (median 1.7%) and to a higher extent pCRT (median 2.1%) significantly increased the percentages of Th17 cells in patients in comparison with HC. In addition, comparing surgery only patients with the cohort of aCRT, in patients who received aCRT after surgery, Th17 frequencies were more elevated (2.7‐fold increase) than in patients with surgery alone.

In contrast to the decrease in absolute numbers of CD4^+^ T cells/µL, numbers of Th17 cells/µL showed no significant differences within the different cohorts (Fig. 1E). Because CD4^+^ T cells comprise Th17 cells, we were further interested in therapy‐induced changes in this CD4^+^ T‐cell subgroup and evaluated the percentage of Th17 per CD4^+^ T cells. The pretherapeutic proportion of Th17 within CD4^+^ T cells of the analyzed cohorts accelerated with cancer progression and correlated with advancing tumor FIGO stages (Fig. S1A; *r* = 0.4830, *P* < 0.0001). During therapy, the cohort of surgery alone showed a significant increase in percentage of Th17 per CD4^+^ T cells in comparison with HC (Fig. 1F; median 1.3% versus 0.7%). Strongest increase we found in patients after aCRT (median 7.8%) and pCRT (10.1%). Patients, who underwent surgery and adjuvant CRT (aCRT), contained significantly more Th17 per CD4^+^ T cells (5.7‐fold increase) in comparison with patients with surgery alone.

Monitoring of patients of the aCRT and pCRT cohorts during therapy demonstrated that these patients exhibited significantly higher pretherapeutic frequencies as well as proportions of Th17 per CD4^+^ T cells in comparison with HC (Fig. S1BC). Patients revealed a slight increase in Th17 frequencies in the aCRT cohort after surgery (Fig. 1G), but a further significant increase in Th17 frequencies (2.3‐fold) took place in 19/20 patients after completion of CRT. In the cohort of pCRT, we found an increase in Th17 frequencies in all 15 analyzed patients after CRT in comparison with beginning of therapy (Fig. 1H, 2.7‐fold increase). Furthermore, all patients showed an increase in percentage of Th17 per CD4^+^ T cells after aCRT (5.6‐fold) or pCRT (4.2‐fold; Fig. 1IJ). Thus, comparing all patients, we found elevated frequencies of Th17 cells and percentages of Th17 per CD4^+^ T cells at varying intensities after different therapeutic approaches. While in patients with surgery alone the Th17 frequency was significantly higher than in HC, aCRT and pCRT strongly increase Th17 frequencies in the blood of cervical cancer patients in comparison with untreated patients or surgery only.

### 3.2 Th17 cells induce resistance of cervical cancer cells toward cisplatin, irradiation, and combined treatment in an IL‐17‐dependent manner

**Fig. 2:**
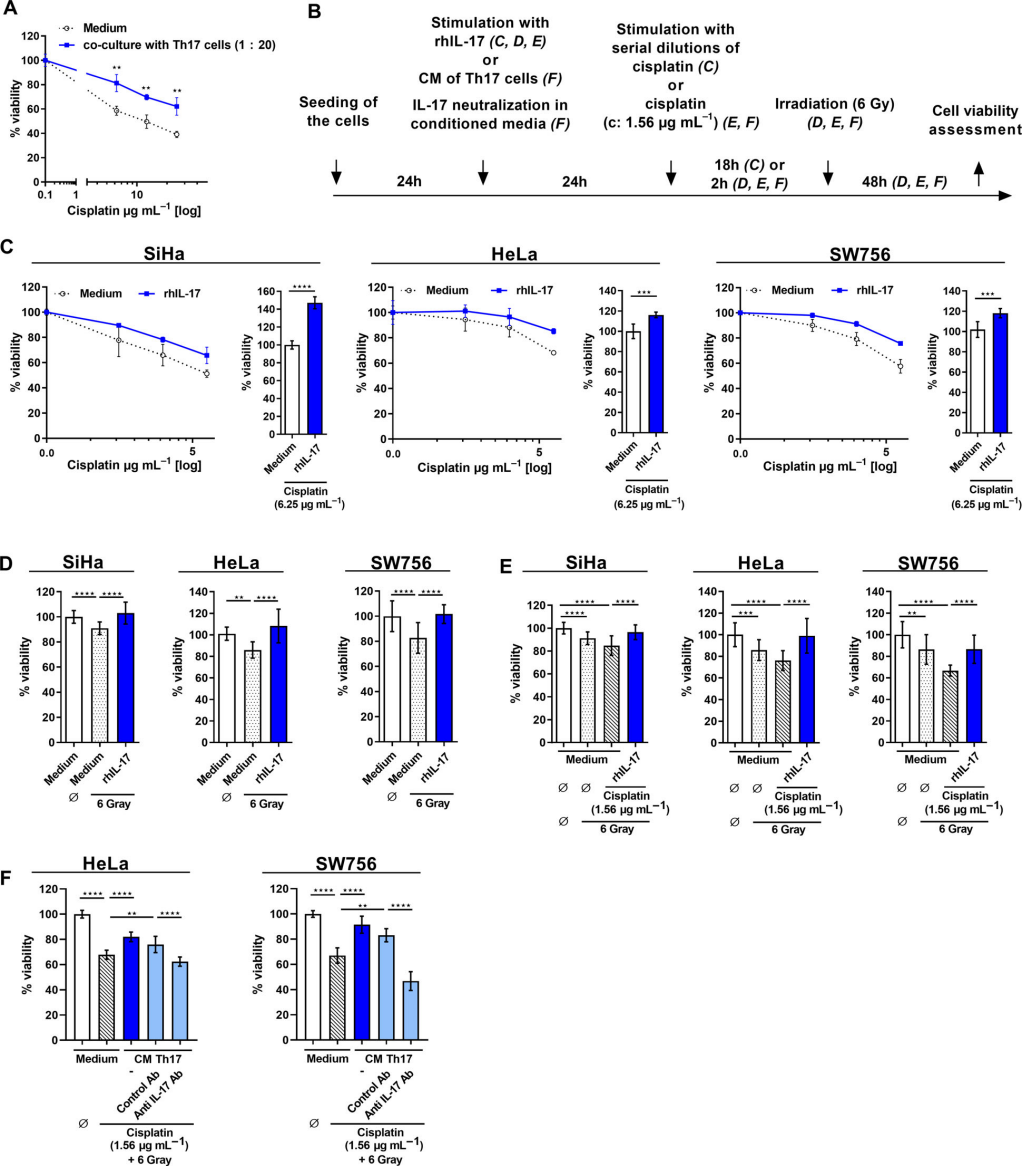
Th17 cells induce resistance of cervical cancer cells toward chemotherapeutic drug cisplatin, irradiation, and combined treatment in an IL‐17‐dependent manner. (A) HeLa cells were cocultured with *in vitro* generated Th17 cells (proportion 1 : 20; blue line) or medium (dotted line). After 24 h, Th17 cells were removed, HeLa cells (both approaches) were washed with PBS and challenged with serial dilutions of cisplatin. After 18 h, cell viability was assessed by the neutral red uptake method. Shown are the results mean ± SD from two independent experiments performed in triplicates. (B) Timeline of the experimental procedures. (C) SiHa (left), HeLa (middle), and SW756 cells (right panel) were stimulated with medium (dotted lines) or rhIL‐17 (blue lines). After 24 h, cells were washed with PBS and challenged with serial dilutions of cisplatin. After 18 h, cell viability was assessed by the neutral red uptake method. Bars represent summary of three independent experiments of the highest cisplatin dose applied. (D, E) SiHa (left), HeLa (middle), and SW756 cells (right) were stimulated with medium (white, dotted, or striped bars) or rhIL‐17 (blue bars) and (D) irradiated with 6 Gy. (E) Cells were treated with 1.56 µg·mL^−1^ cisplatin for 2 h and irradiated with 6 Gy. (F) HeLa (left) and SW756 cells (right) were stimulated with medium (white or striped bars) or CM of *in vitro* generated Th17 cells (blue bars). In neutralization experiments, CM were prestimulated with neutralizing anti‐IL‐17 or respective isotype control antibodies for 2 h (light blue bars). Cells were treated with 1.56 µg·mL^−1^ cisplatin for 2 h and irradiated with 6 Gy. After 48 h, cell viability was assessed by the neutral red uptake method. Shown are the results mean ± SD from three independent experiments performed in triplicates. *P*‐value according to the nonparametric Mann–Whitney U‐test (A, C) or Kruskal–Wallis test (D, E, F). Asterisks represent statistical significances: **P < 0.01; ***P < 0.001; ****P < 0.0001

Since Th17 cells infiltrate cervical cancer tissues and their presence *in situ* correlated with progression of the disease [13, 21], we were interested whether Th17 cells influence responsiveness of cervical cancer cells toward chemotherapeutic drug or irradiation. First, we tested cisplatin, the most common drug used in cervical cancer chemotherapy. We cocultivated HeLa cells with *in vitro* generated Th17 cells at a ratio of 1:20 for 24 h, removed the Th17 cells, and challenged the cervical cancer cells with serial dilutions of cisplatin. As a consequence, cocultivation decreased the responsiveness of HeLa cells toward cisplatin‐induced cell death (Fig. 2A) resulting in up to 23% increased cell viability.

Next, we analyzed whether cell death resistance toward cisplatin was mediated by direct cell–cell contact or whether soluble factors produced by Th17 cells were sufficient (schematic procedure of the following experiments in Fig. 2B). The applied cisplatin doses killed the individual cell lines SiHa, HeLa, or SW756 from 74 to 55% (Fig. 2C, dotted lines). As a uniform response in all tested cancer cell lines, prestimulation of SiHa, HeLa, and SW756 cells (Fig. 2C) with rhIL‐17A (blue lines and bars) significantly increased cell viability after cisplatin treatment reaching from 10 to 18% at the highest applied cisplatin doses of 6.25 µg·mL^−1^ (Fig. 2C, respective bar graphs). Same results we obtained in irradiation experiments in which we used a dose of 6 Gy, resulting in highest cell death sensitization in previous experiments (Fig. S2). Treatment with 6 Gy reduced cell viability from 10 to 18% in all three tested cells (Fig. 2D; dotted bars) and prestimulation of the cells with rhIL‐17 (blue bars) significantly increased cell viability after irradiation (13‐32% increase). Since CCRT is the gold standard in treatment of cervical cancers > FIGO IIB [1], we combined cisplatin treatment and irradiation to mimic chemoradiotherapy *in vitro*. Chemoradiotherapy further decreased cell viability (Fig. 2E, striped bars; 16‐34% decrease) in all three cell lines. Again, pretreatment with recombinant IL‐17 (blue bars) significantly reduced the responsiveness of the cells toward chemoradiotherapy, resulting in 12‐27% increased cell viability. Finally, we analyzed the impact of CM of *in vitro* generated Th17 cells on HeLa and SW756 cells showing highest sensitivity toward chemoradiotherapy *in vitro* (Fig. 2E). Prestimulation of HeLa and SW756 cells (Fig. 2F) with CM (blue bars) reduced the responsiveness toward chemoradiotherapy‐induced cell death, resulting in 15–24% increase in cell viability. Neutralization of IL‐17 in the CM of Th17 cells completely abolished Th17‐induced resistance (Fig. 2F, light blue bars). Taken together, our results clearly showed that IL‐17 is the responsible factor in the CM of Th17 cells for chemoradiotherapy resistance of cervical cancer cells.

### 3.3 Th17‐induced resistance of cervical cancer cells is AKT‐dependent

**Fig. 3:**
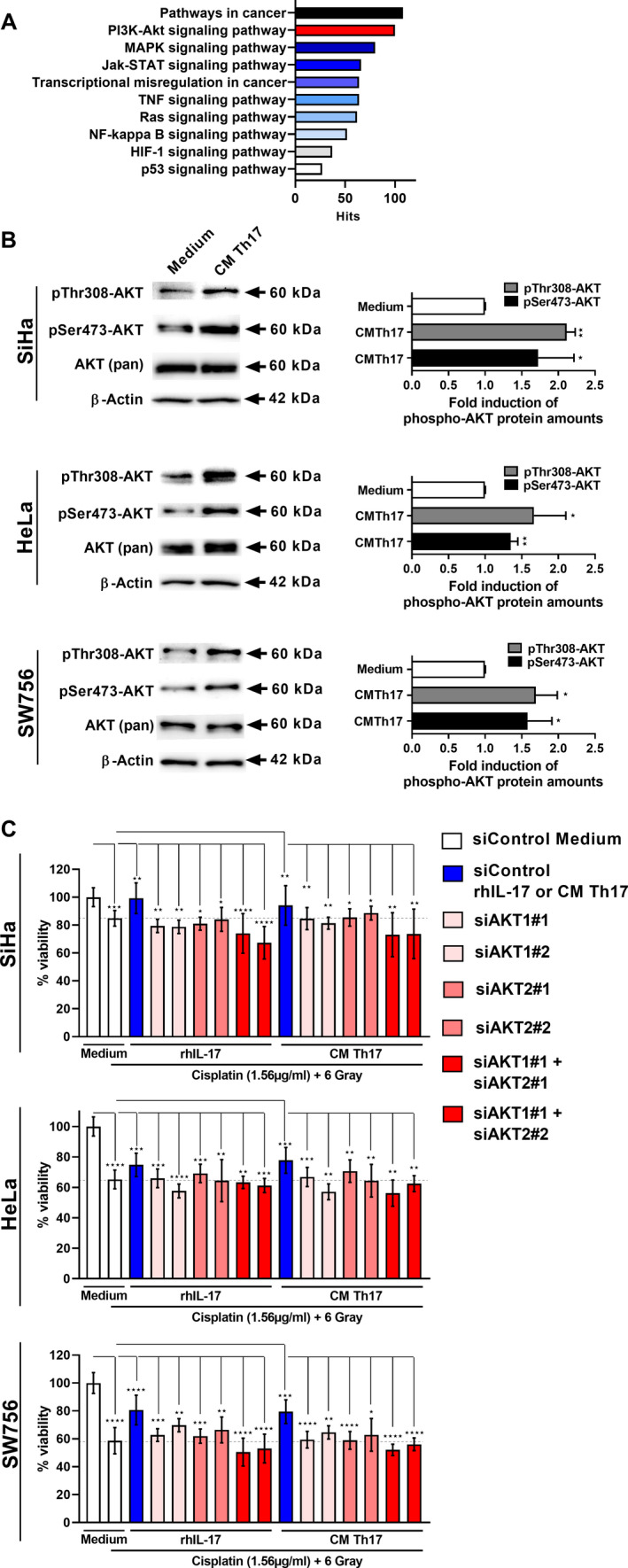
Th17‐induced resistance of cervical cancer cells is AKT‐dependent. Over‐representation analysis of 5639 mRNAs, which were at least 1.5‐fold induced in SiHa or SW756 cells after stimulation with CM of *in vitro* generated Th17 cells by GeneTrail2. (A) Representative significant enriched pathways in KEGG (Kyoto Encyclopedia of Genes and Genomes). (B) SiHa (top), HeLa (middle), and SW756 (down) were stimulated with medium or CM of *in vitro* generated Th17 cells. After 15 min, whole cell extracts were analyzed for pThr308‐AKT, pSer473‐AKT, and AKT (pan) expression by western blot analysis. β‐Actin was used as a loading control. Shown is one representative experiment out of three independent experiments. Bars represent quantification of three independent experiments. (C) SiHa (top), HeLa (middle), and SW756 cells (down) were transfected with two specific siRNAs for AKT1 (light red bars) or AKT2 (middle red bars), respectively, both isoforms (dark red bars) or mock siRNA (white and blue bars) as a control and stimulated with medium, rhIL‐17 or CM of Th17 cells (blue bars). Cells were incubated with 1.56 µg·mL^−1^ cisplatin for 2 h and irradiated with 6 Gy. After 48 h, cell viability was assessed by the neutral red uptake method. Shown are the results mean ± SD from three independent experiments performed in triplicates. Gray dotted lines mark levels of sensitivity toward chemoradiotherapy of the respective unstimulated cells. *P*‐value according to the nonparametric Mann–Whitney U‐test or Kruskal–Wallis test. Asterisks represent statistical significances: *P < 0.05; **P < 0.01; ***P < 0.001; ****P < 0.0001.

Next, we were interested in the mechanism responsible for Th17‐induced resistance and performed a mRNA expression analysis (Agilent SurePrint G3 Human GE v3 8x60K microarray) of SiHa and SW756 cells after stimulation with CM of *in vitro* generated Th17 cells. Out of 58000 detectable mRNAs of the microarray, 5639 protein coding transcripts were at least 1.5‐fold changed after CM stimulation in comparison with medium stimulated control cells. To arrange the at least 1.5‐fold changed protein coding transcripts in pathways, we used GeneTrail2 (https://genetrail2.bioinf.uni‐sb.de/) [26]. Performing an over‐representation analysis (ORA), we identified 2869 significant pathways (*P* ≤ 0.05) which belonged to Gene Ontology, Wiki Pathways, or KEGG (Kyoto Encyclopedia of Genes and Genomes). We analyzed all subcategories for cancer‐related pathways and found a significant enrichment of the 1.5‐fold changed mRNAs in 217 pathways of KEGG. Fig. 3A displays a representative selection of 10 significant pathways of the KEGG category with highest enrichment in the subcategories ‘Pathways in cancer’ (108 genes), ‘PI3K‐AKT signaling pathway’ (100 genes), ‘MAPK signaling pathway’ (80 genes), and ‘Jak‐STAT signaling pathway’ (66 genes).

We analyzed the impact of the highest enriched pathways (Jak‐STAT, MAPK, and PI3K‐AKT signaling) for Th17‐induced resistance toward chemoradiotherapy in cervical cancer cells. The transcription factor STAT3 is considered as a survival or progression factor in different cancer types affecting the response of certain tumors to chemotherapy [27]. In line with our previous results, STAT3 activation was weak or absent in cervical cancer cells ([25] and Fig. S3A). Stimulation with rhIL‐17 mediated only weak pSTAT3(Tyr705) expression in comparison with the known STAT3 activator Oncostatin M (OSM) in three different cervical cancer cell lines (Fig. S3A). Furthermore, knock down of MAPK1 or MAPK14 with two respective specific siRNAs did not significantly affect IL‐17‐induced resistance toward chemoradiotherapy in three tested cervical cancer cells (Fig. S3BC).

To verify the activation of the AKT signaling pathway, we stimulated cervical cancer cells with CM of *in vitro* generated Th17 cells and analyzed the pThr308‐ and pSer473‐AKT expression (Fig. 3B). Phosphorylation on threonine and serine residues is required to obtain full AKT enzymatic activity [28]. Stimulation revealed a significant increase in pThr308‐ (up to 2.1‐fold) and pSer473‐AKT expression (up to 1.7‐fold) in all three tested cell lines. AKT comprises three distinct isoforms (AKT1‐3). In line with previous results, HeLa cells lack AKT3 expression ( [29] and Fig. S4A). Same results we obtained for SiHa cells while in SW756 cells AKT3 expression was detectable. AKT1 and AKT2 expression was present in all three cervical cancer cell lines (Fig. S4BCD). To analyze the influence of AKT signaling in Th17‐induced resistance toward chemoradiotherapy, we established knock downs of the ubiquitously expressed isoforms AKT1 and AKT2. This resulted in 82‐97% knock down of AKT1 and 82‐95% knock down of AKT2 in western blot analysis using AKT1‐ and AKT2‐specific antibodies (Fig. S4BCD). We knocked down AKT1, AKT2, or both together (Fig. 3C), stimulated the cells with rhIL‐17 or CM of Th17 cells, and used the cells in chemoradiotherapy experiments. In all three cell lines, knock down of AKT1 (light red bars) and AKT2 (middle red bars) significantly reduced IL‐17‐ or CM‐induced cell death resistance toward chemoradiotherapy. Complete reversion was obtained after simultaneous knock down of AKT1 and AKT2 (dark red bars). This was also the case in SW756 cells expressing AKT3, indicating that AKT3 is not involved in Th17‐induced resistance. In line with this, CaSki cells with PIK3CA mutation resulting in constitutively activated AKT [30] showed no responsiveness toward irradiation. While chemoradiotherapy only slightly reduced cell viability (10% decrease) prestimulation with rhIL‐17 as well as CM of Th17 cells completely reverted cell viability (Fig. S4E). These data provided evidence that Th17‐induced resistance toward combined treatment with cisplatin and irradiation depends on the AKT pathway in cervical cancer cells.

### 3.4 Th17 cells correlate with pThr308‐ and pSer473‐AKT expression in cervical cancers *in situ* and are associated with response to primary chemoradiotherapy in cervical cancer patients

**Fig. 4:**
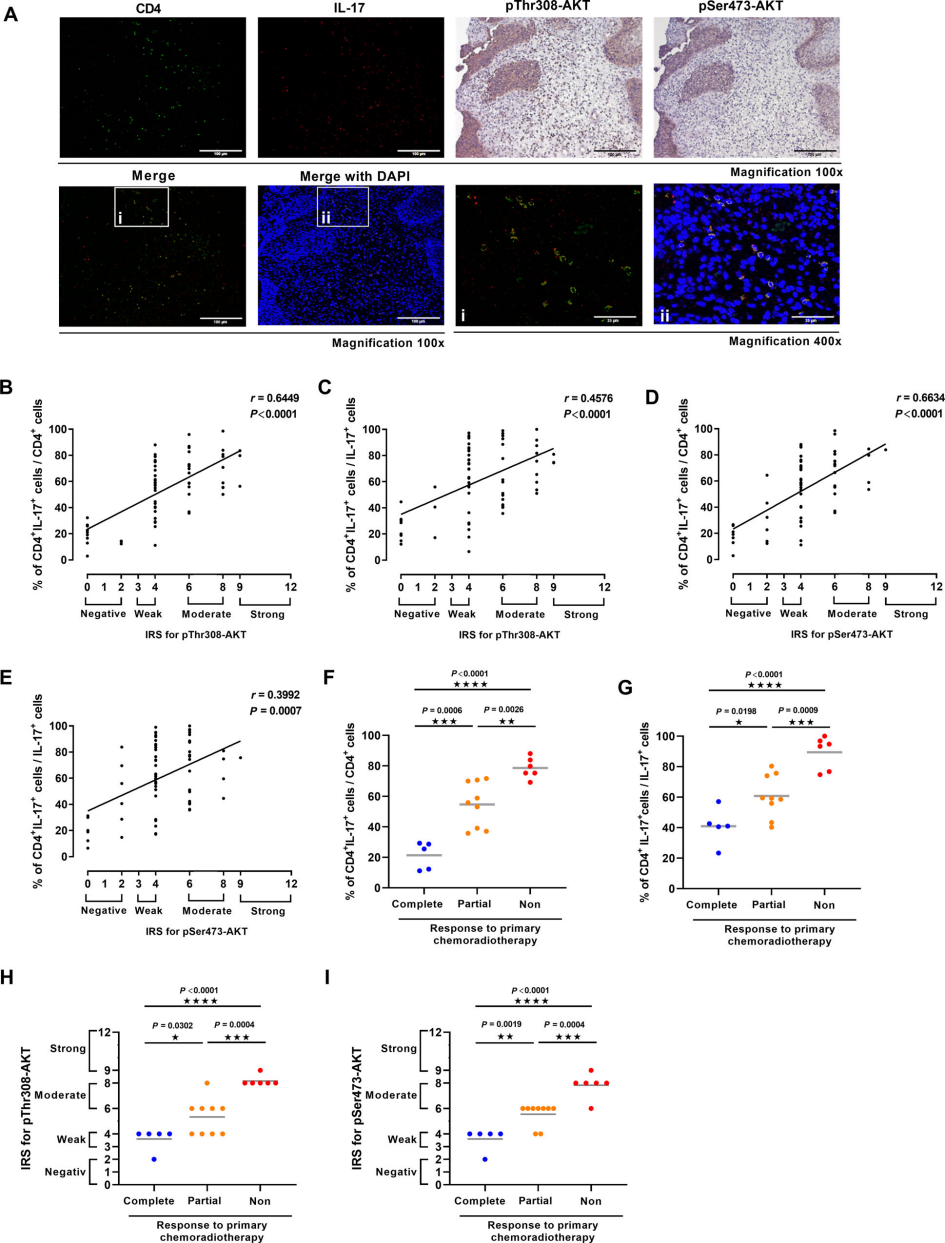
Th17 cells correlated with pThr308‐ and pSer473‐AKT expression in cervical cancer biopsies *in situ* and are associated with response to primary chemoradiotherapy in cervical cancer patients. (A) Sections of human SCCs of 70 cervical cancer patients were stained for pThr308‐ and pSer473‐AKT expression by IHC or costained for CD4 (green), and IL‐17 (red) by immunofluorescence. Magnification 100× (Scale bars 100 µm), magnification 400× (Scale bars 25 µm). (B, D) The percentage of CD4^+^IL‐17^+^ cells per CD4^+^ cells correlated with (B) the IRS of pThr308‐AKT or (D) the IRS of pSer473‐AKT. (C, E) The percentage of CD4^+^IL‐17^+^ cells per IL‐17^+^ cells correlated with (C) the IRS of pThr308‐AKT or (E) the IRS of pSer473‐AKT. (F, G, H, I) The percentage of CD4^+^IL‐17^+^ cells per CD4^+^ cells, the percentage of CD4^+^IL‐17^+^ cells per IL‐17^+^ cells, IRS of pThr308‐ and pSer473‐AKT expression were determined in pretherapeutic biopsies of *n* = 20 cervical cancers and compared with the individual patient's response (non (red circles), partial (orange circles), or complete response (blue circles)) to primary chemoradiotherapy. *P*‐value according to the nonparametric Kruskal–Wallis test (F, G, H, I) or Spearman rank correlation with linear regression (B, C, D, E). Asterisks represent statistical significances: *P < 0.05; **P < 0.01; ***P < 0.001; ****P < 0.0001.

To investigate the relationship between Th17 cells and activation of AKT signaling in cervical cancer patients, we analyzed the presence of Th17 cells by IF and the pThr308‐ and pSer473‐AKT expression by IHC (representative pictures in Fig. 4A). Cervical cancer biopsies of 70 analyzed patients were differentially infiltrated by CD4^+^ and IL‐17^+^ cells which significantly correlated with numbers of CD4^+^IL‐17^+^ (Th17) cells·mm^−2^ (Fig. S5AB) whereas no correlation was found between the numbers of CD4^−^/IL‐17^+^ cells and IL‐17^+^ cells·mm^−2^ (Fig. S5C). Since Th17 cells represent a subpopulation of CD4^+^ T cells and a part of IL‐17‐expressing cells we evaluated the percentages of CD4^+^IL‐17^+^ (Th17) cells per total infiltrating CD4^+^ or IL‐17^+^ cells. Interestingly, in biopsies of patients with lymph node metastases (Fig. S5D, left) or patients who retrospectively developed relapse (Fig. S5D, right), more CD4^+^ or IL‐17^+^ cells represent Th17 cells in comparison with patients without metastases or relapse. Importantly, the percentage of Th17 cells per total CD4^+^ or IL‐17^+^ cells significantly correlated with heterogeneous pThr308‐AKT and pSer473‐AKT expression *in situ* reaching from negative to strong after applying the IRS (Fig. 4B‐E; Fig. S6A) indicating a functional link between Th17 cells and AKT signaling *in vivo*. We could previously show that the numbers of Th17 cells *in situ* correlated with enhanced tumor FIGO stages [13]. Here, we found that more advanced cervical cancers, patients with lymph node metastases or who retrospectively developed cervical cancer recurrence exhibited stronger AKT phosphorylation *in situ* (Fig. S6C‐H). Thus, the results from our *in situ* analysis indicated that the proportions of Th17 within CD4^+^ or IL‐17^+^ cells might be linked to enhanced activation of AKT signaling pathway during cervical cancer progression.

We then evaluated the presence of Th17 cells, pThr308‐ and pSer473‐AKT expression in 20 human cervical cancer biopsies prior to primary chemoradiotherapy in relation to the patient's individual response to therapy (Supplementary Table S1). This cohort of patients showed a significant correlation between the percentage of Th17 per CD4^+^ T cells and pThr308‐ or pSer473‐AKT expression as well as between pThr308‐ and pSer473‐AKT expression *in situ* (Fig. S6IJK). Notably, patients with no response to chemoradiotherapy (red circles) displayed significantly higher proportions of Th17 cells in their tumor tissues (Fig. 4FG; *P* = 0.0026, *P* = 0.0009) and higher scores of pretherapeutic pThr308‐ (Fig. 4H; *P* = 0.0004) or pSer473‐AKT expression (Fig. 4I; *P* = 0.0004) in neoplastic cells than patients with only partial response to therapy (orange circles). Patients with complete response to therapy (blue circles) showed lowest frequencies of Th17 cells and pretherapeutic pThr308‐ or pSer473‐AKT expression. These data demonstrate a clear association between pretreatment frequencies of Th17 cells as well as pThr308‐ and pSer473‐AKT expression and response to chemoradiotherapy *in vivo*.

### 3.5 Increased Th17 frequencies after chemoradiotherapy in the blood of cervical cancer patients are associated with recurrent cervical cancers

**Fig. 5:**
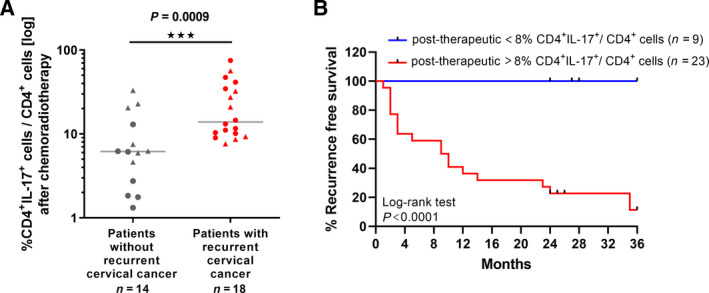
Increased Th17 frequencies after chemoradiotherapy in the blood of cervical cancer patients were associated with recurrent cervical cancers. (A) The percentage of CD4^+^IL‐17^+^ cells per CD4^+^ T cells of 32 patients which received aCRT (circles, *n* = 18) or pCRT (triangles, *n* = 14) was evaluated. The percentage of CD4^+^IL‐17^+^ cells per CD4^+^ T cells was depicted for patients with recurrent cervical cancers (*n* = 18; red circles and triangles) in comparison with patients without relapse (*n* = 14; gray circles and triangles). *P*‐value according to the nonparametric Mann–Whitney *U*‐test. Asterisks represent statistical significances: ***P < 0.001. (B) Recurrence‐free survival of 32 patients (*n* = 18 after aCRT, *n* = 14 after pCRT) was determined for a cohort with CD4^+^IL‐17^+^ cells per CD4^+^ T cells < 8% after therapy (blue line) in comparison with a cohort of post‐therapeutic CD4^+^IL‐17^+^ cells per CD4^+^ T cells > 8% (red line). Median recurrence‐free survival was 9 months for the cohort of CD4^+^IL‐17^+^ cells per CD4^+^ T cells > 8%. Comparison of survival analysis was performed using log‐rank (Mantel–Cox) test; chi‐square: 15.22, *P* < 0.0001.

To clarify the impact of increased proportions of Th17 cells in the blood of patients after aCRT and pCRT for clinical outcome, 32 patients (with follow‐up times of at least 24 months) out of 35 analyzed patients after CRT could be evaluated for cervical cancer recurrence. The median follow‐up over all was 29 months (range 24–36 months). Retrospectively, 18/32 patients developed cervical cancer recurrence (Fig. 5A). Patients with cancer relapse exhibited slightly, but not significantly enhanced tumor FIGO stages (Fig. S7A). Patients with recurrent cervical cancers showed significant 2.2‐fold higher amounts of Th17 cells per CD4^+^ T cells after therapy in their blood (median 13.89%; *P* = 0.0009) in comparison with patients without relapse (median 6.2%; Fig. 5A) regardless whether they were treated with aCRT (circles) or pCRT (triangles). Both therapeutic approaches resulted in comparable proportions of Th17 per CD4^+^ T cells in the patients` blood (Fig. 1F). Patients with and without cervical cancer recurrence did not differ in pretherapeutic Th17 frequencies and proportions of Th17 cells per CD4^+^ T cells in their blood (Fig. S7BC).

To identify a cutoff value concerning the frequency of Th17 cells in the blood after therapy, which discriminates between patients with and without recurrent cervical cancers, ROC analyses were performed. Best discrimination was obtained for a cutoff value of Th17 per CD4^+^ T cells > 8% with 100% sensitivity and 71.43% specificity. The area under the ROC curve (AUC) value was 0.8333. Applying the defined cutoff for 32 patients after CRT, in the group of patients with percentages of Th17 per CD4^+^ T cells < 8% all patients showed recurrence‐free survival in Kaplan–Meier curves (Fig. 5B, blue line). In contrast, in the group of patients with percentages of Th17 per CD4^+^ T cells > 8%, 2‐ and 3‐year recurrence‐free survival was 22.7% and 11.4% (red line). In conclusion, our data demonstrated a clear association between post‐therapeutic Th17 cell levels and cervical cancer recurrence.

## 4 Discussion

**Fig. 6:**
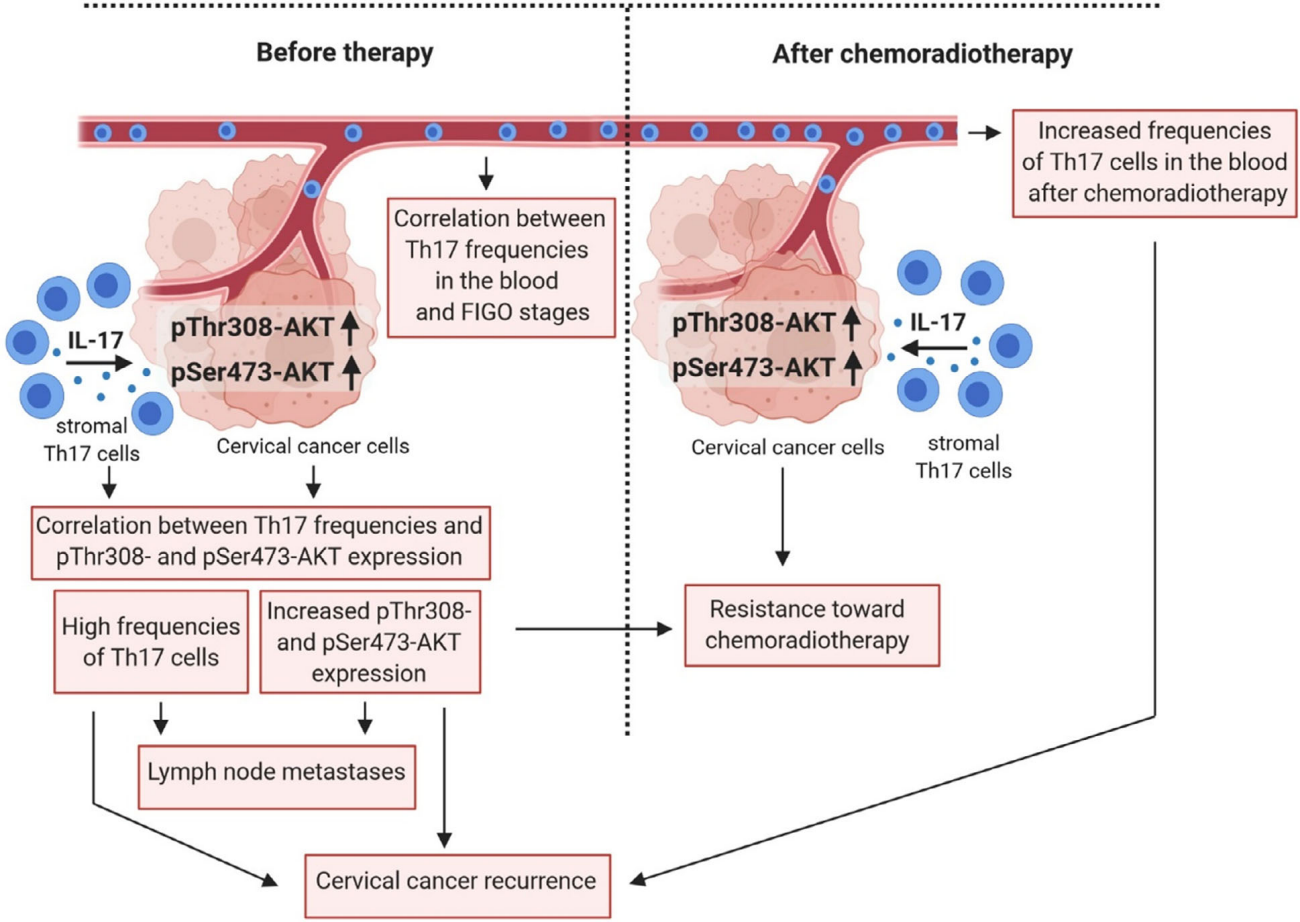
Schematic presentation of the dual role of Th17 cells in cervical cancer therapy: influence of Th17 cells on cervical cancer cells and increased frequencies of Th17 cells after chemoradiotherapy related to AKT‐dependent resistance and cervical cancer recurrence (scheme was generated with the BioRender software).

Th17 cells, an IL‐17‐expressing T‐cell subset with protumorigenic properties, infiltrate cervical cancers and correlate with advanced tumor stages [13, 21] and progression of the disease [14]. In this study, we investigated Th17 cells in cervical cancer stage‐dependent therapy. On the one hand, we analyzed the impact of different therapy approaches such as surgery, aCRT in addition to surgery and pCRT alone on Th17 frequencies in the blood of cervical cancer patients. On the other hand, we examined the influence of Th17 cells on the responsiveness of cervical cancer cells toward cisplatin, irradiation, and combined treatment. We could show that frequencies of Th17 cells increased during CRT in the blood of cervical cancer patients. Notably, enhanced proportions of Th17 cells in patients after aCRT as well as pCRT were associated with recurrent cervical cancers. Our study has unraveled a key role of Th17‐induced AKT signaling in cervical cancer cells for resistance toward chemoradiotherapy. Our data demonstrated a significant correlation between Th17 cells and AKT activation *in situ* and showed that cervical cancer patients with higher frequencies of Th17 cells and phosphorylated AKT expression scores in their tumor tissues showed a significantly lower response to CRT. Fig. 6 summarizes our current concept of our findings concerning the dual role of Th17 cells in cervical cancer therapy, which may have major implications for personalization of immunotherapy.

The HPV‐associated tumor microenvironment is critical for tumor development and progression. While the presence of Th1 cells and M1 macrophages has been positively correlated with clinical outcome [3, 31], tumor‐infiltrating M2 macrophages, myeloid‐derived suppressor cells (MDSCs), or regulatory T cells (Tregs) have been associated with lymph node metastases [32] and favor an immune‐suppressive and tumor‐promoting environment [33]. Effects of oncological therapies on leucocytes or lymphocyte subsets have been of extensive interest, because of the possibility that therapy‐induced changes in immune‐cell homeostasis might interfere with antitumor activity supporting therapy resistance. A study demonstrated an increase in myeloid cell populations, such as immune‐suppressive monocytic MDSCs, after (chemo)radiotherapy in the blood of thirty cervical cancer patients [3]. Th17 cells were described as long living effector cells with high proliferative capacity and apoptosis‐resistant phenotype toward different cell death inducers, such as the chemotherapeutic drug cisplatin [4], Fas‐mediated cell death in murine Th17 cells [34], or transarterial chemoembolization in hepatocellular carcinoma patients [35]. In this study, monitoring of cervical cancer patients during cervical cancer therapy showed a decrease in CD4^+^ T cells in the patients` blood, but a significant increase in Th17 frequencies after aCRT and pCRT in comparison to patients with surgery alone. Analysis of occurrence of Th17 cells within the remaining CD4^+^ T cells revealed enhanced proportions of Th17 per CD4^+^ cells. This accumulation was especially magnified in the CRT patient cohorts, implying that Th17 are resistant to CRT. This is in line with the findings that human Th17 cells showed upregulation of anti‐apoptotic genes after irradiation *ex vivo* [36]. Strikingly, our data show an association between enhanced Th17 frequencies after therapy and cervical cancer recurrence. In the cohort of patients with proportions of Th17 per CD4^+^ T cells >8% after therapy, the 3‐year recurrence‐free survival was only 11.4%.

In contrast to other tumor entities with IL‐17‐expressing tumor cells, IL‐17‐producing cells in cervical cancer tissues were predominantly found in the stroma [13, 37]. IL‐17 expressing neutrophils, mast cells, or γδT cells have been found in different cancers potentially linked to poor prognosis [38]. Our *in situ* analysis demonstrated that high occurrence of Th17 cells in cervical cancer tissues is associated with cancer progression ([13] and this study). Because of the variable numbers of infiltrating CD4^+^ or IL‐17^+^ cells per patient`s biopsies and the high plasticity of the CD4^+^ T cell or IL‐17^+^ cell compartment, evaluation of the proportions of Th17 cells per infiltrating CD4^+^ or IL‐17^+^ cells can clarify their distributions within the individually present CD4^+^ or IL‐17^+^ cells in cancer tissues. IL‐17 expression was not solely found in T cells in cervical cancers ([37] and this study); however, our analysis revealed that in tumor tissues of patients with lymph node metastases or who retrospectively developed cervical cancer recurrence more CD4^+^ or IL‐17^+^ cells represent Th17 cells indicated by higher percentages of Th17 cells per total CD4^+^ or IL‐17^+^ cells. The presence of tumor‐infiltrating stromal Th17 cells in cervical cancer tissues might affect therapeutic approaches. Analyzing the impact of Th17 cells on cervical cancer cells, we found that Th17 cells reduced the responsiveness of cervical cancer cells toward cisplatin, irradiation, and combined treatment in an IL‐17‐dependent manner. As a responsible resistance mechanism, we identified Th17‐induced AKT signaling while STAT3 or MAPK activation was not involved. Particularly, STAT3 activation in patients biopsies *in situ* declines during cancer progression from preinvasive to invasive cancers in which pSTAT3(Tyr705) expression was low or absent [25]. The serine/threonine kinase AKT comprises three distinct isoforms (AKT1‐3). To obtain full enzymatic activity, phosphorylation on threonine and serine residues is required [28]. In our experiments, knock down of AKT1 and AKT2 completely reverted Th17‐induced resistance in AKT3‐negative cervical cancer cell lines SiHa and HeLa and also in the AKT3 expressing cell line SW756, indicating that the more ubiquitously expressed isoforms AKT1 and AKT2 were sufficient for Th17‐induced resistance. AKT is part of the phosphoinositide 3‐kinase (PI3K)/AKT signaling cascade and crucial in tumor development promoting proliferation and anti‐apoptotic as well as pro‐angiogenic effects [28]. PIK3CA mutations resulting in aberrant AKT activation in cervical cancer patients have been associated with worse prognosis after chemoradiotherapy [39]. In line with this, cervical cancer cells with PIK3CA mutations and increased AKT activation, such as CaSki cells [30], exhibited lowest responsiveness toward (chemo)radiotherapy. All used cervical cancer cells of this study showed highest resistance toward therapy under impact of Th17 cells. In other tumor entities, tumor cell‐ or stromal cell‐derived IL‐17 induces AKT signaling which is involved in cisplatin resistance of colorectal cancer [40] or EMT, invasion, and metastasis in hepatocellular carcinoma [41]. Thus, PI3K/AKT signaling represents an attractive target for several clinical trials [42, 43].

As Th17‐induced AKT activation was sufficient to render cervical cancer cells more resistant toward different therapeutic approaches, we were interested in the *in vivo* situation. Activation of the AKT pathway is associated with cervical cancer progression [44] and reduced progression‐free survival in patients after irradiation [45] or chemoradiotherapy [46]. In line with this, we found an increased threonine 308 und serine 473 phosphorylation *in situ* during cancer progression based on tumor FIGO stages and patients with lymph node metastases or who retrospectively developed cervical cancer relapse showed significantly increased pThr308‐ and pSer473‐AKT expression in their tumor tissues. Notably, in the analyzed patient samples, Th17 frequencies significantly correlated with pThr308‐ and pSer473‐AKT expression. This was interesting, as AKT signaling can also be induced by mediators other than Th17 cells, such as PI3K mutations [47], EGFR signaling [48], or hypoxia [29, 49]. Particularly, hypoxia‐induced AKT signaling repressed the expression of the HPV oncogenes E6 and E7 in cervical cancer cells and allowed hypoxic cells to escape from chemotherapeutic drug‐induced senescence [50]. Furthermore, hypoxia promotes the differentiation of Th17 cells [51] favoring the idea of potential synergistic mechanisms of hypoxia and Th17 cells regarding AKT activation and therapy resistance in cervical cancers. Importantly, we found a close connection between the presence of Th17 cells and AKT activation *in situ* and therapy response. We detected the highest Th17 amounts, pThr308‐ and pSer473‐AKT pretreatment expression scores in cancers of those patients who did not respond to pCRT. In contrast, partial or complete responders displayed significantly lower Th17 amounts and phosphorylated AKT expression scores.

For patients who do not respond to standard treatments or immunotherapies, new strategies are needed. In patients with advanced, metastatic or recurrent cervical cancer immunotherapies such as bevacizumab, an anti‐VEGF antibody to inhibit angiogenesis [52, 53], and pembrolizumab, an anti‐PD1 antibody [54], are approved as targeted therapies in combination with a platinum‐based chemotherapy. In our study, pretherapeutic Th17 frequencies in the patients` blood did not significantly discriminate between patients with or without cancer relapse. Clinically most important, here we identified an association between elevated Th17 frequencies after CRT in the patients` blood and the course of disease. 18 of 32 patients after aCRT and pCRT with elevated Th17 frequencies after therapy developed cancer recurrence within 3 years, suggesting that the imbalance of the immune milieu might promote disease progression. Patients with cancer relapse exhibited more advanced tumors based on FIGO stages. However, FIGO classifications did not significantly distinguish between patients with or without cancer recurrence. Best discrimination between patients with and without cancer relapse was obtained using a cutoff value of post‐therapeutic Th17 per CD4^+^ T cells > 8%. In this cohort of patients, recurrence‐free survival was strongly decreased exhibiting only 11.4%, while in the group of patients with percentages of Th17 per CD4+ T cells < 8% recurrence‐free survival was 100%. In line with our results, one group demonstrated just now that an increase in circulating Th17 cells after pCRT in Chinese cervical cancer patients was associated with reduced therapy response rate and progression‐free survival. However, in contrast to our results this study also found patients with decreased Th17 frequencies after pCRT [55]. Whether the results of this study are due to ethnical, environmental, or technical issues remains to be determined. In addition to patients with pCRT, we analyzed frequencies of Th17 cells during different therapeutic approaches and found increased Th17 cells after aCRT, too, indicating that Th17 cells might favor tumor‐promoting events before, during, and after CRT which may support metastases. The results of our study may have major implications for personalization of cervical cancer therapy. On the basis of our results, pretreatment Th17 numbers *in situ* should be evaluated as a predictive biomarker for the individual response of cervical cancer patients to CRT or target for immunotherapy in prospective clinical studies. Furthermore, phenotypic and functional characterization of chemoradiotherapy‐induced IL‐17‐producing CD4^+^ T cells in the blood of larger patients cohorts would be of great interest to clarify the mechanisms responsible for Th17 resistance toward chemoradiotherapy and Th17‐based correlation with cancer relapse. Antibodies against IL‐17A or IL‐17 receptor are approved for the treatment of psoriasis [56] and are currently being evaluated for treatment of inflammatory diseases [57] and different cancers [58, 59, 60] and should also be considered for cervical cancer therapy, potentially in combination with chemoradiotherapy.

## 5 Conclusions

Our study identified a novel role of Th17 cells in cervical cancer therapy. We could clarify that Th17 cells act in a dual way during CRT. First, they mediated AKT‐dependent resistance of cancer cells and high pretherapeutic Th17 scores in cancer biopsies were associated with lower treatment responses of patients to pCRT. Second, Th17 cells remained in the patients` blood after CRT and elevated post‐therapeutic Th17 frequencies refer to early cancer relapse. Our results may help to explain therapy resistance and individual treatment responses after CRT of cervical cancer patients and favor the idea of Th17‐based immunotherapeutic approaches in cancer treatments.

## Conflict of interest

The authors declare that they have no competing interests.

### Peer Review

The peer review history for this article is available at https://publons.com/publon/10.1002/1878‐0261.13095.

## Author contributions

RS, PM, and BWR designed the study; LT and BWR designed the experiments; LT, TT, MP, BG, and BWR developed methodology and performed experiments; RS, III, PM, SM, DS, CH, MH, NL, EM., YJK, RMB, SS, CR, EFS and BWR contributed to study design, patient recruitment, technical and material support and clinical data acquisition. LT, RS, PM, EFS and BWR contributed to analysis and interpretation of data. YJK contributed to conducting, analysis, and interpretation of histopathologic studies. LT and BWR supervised all parts of the study and wrote the original draft of the manuscript. LT, RS, PM, III, MP, SM, DS, MH, EM, YJK; SS and BWR involved in review and editing of the manuscript. All authors approved the final version of the manuscript.

## Supporting information


**Fig. S1**. Pre‐therapeutic frequencies of Th17 cells in the patients` blood correlated with tumor FIGO stages.

**Fig. S2**. Cervical cancer cells exhibit different radiosensitivity.

**Fig. S3**. Impact of rhIL‐17 on the activation of STAT3 signaling and role of MAPK1 and MAPK14 on the IL‐17‐mediated resistance toward chemoradiotherapy in cervical cancer cells.

**Fig. S4**. Analysis of AKT3 expression in different cervical cancer cells and specific knock down of AKT1 and AKT2.

**Fig. S5**. CD4+IL‐17+ cells infiltrate cervical SSCs and correlate with numbers of CD4+ and IL‐17+ cells, lymph node metastases and recurrent cervical cancers.

**Fig. S6**. pThr308‐ and pSer473‐AKT expression in cervical cancer biopsies in situ correlate with cancer progression and Th17 cells.

**Fig. S7**. FIGO stages as well as pre‐therapeutic Th17 frequencies did not significantly discriminate between patients with or without cervical cancer relapse.

**Table S1**. Pre‐ and post‐therapeutical stages according to the International Federation of Gynecology and Obstetrics (FIGO) or TNM categories.

**Table S2**. Immunoreactive Score (IRS) according to Remmele & Stegner.

**Table S3**. List of used materials.

## Data Availability

The data supporting the conclusions of this article are included within the article and its additional files.
