# Supplementary material for: Chemoradiotherapy‐induced increase in Th17 cell frequency in cervical cancer patients is associated with therapy resistance and early relapse
Source: Mol Oncol. 2021 Sep 13;15(12):3559–77. doi: 10.1002/1878-0261.13095 (PMC8637579; doi:10.1002/1878-0261.13095)
Supplement: Supplementary file 1 — Fig. S1. Pre‐therapeutic frequencies of Th17 cells in the patients` blood correlated with tumor FIGO stages. [file MOL2-15-3559-s003.pdf]

## Supplementary Figure S1

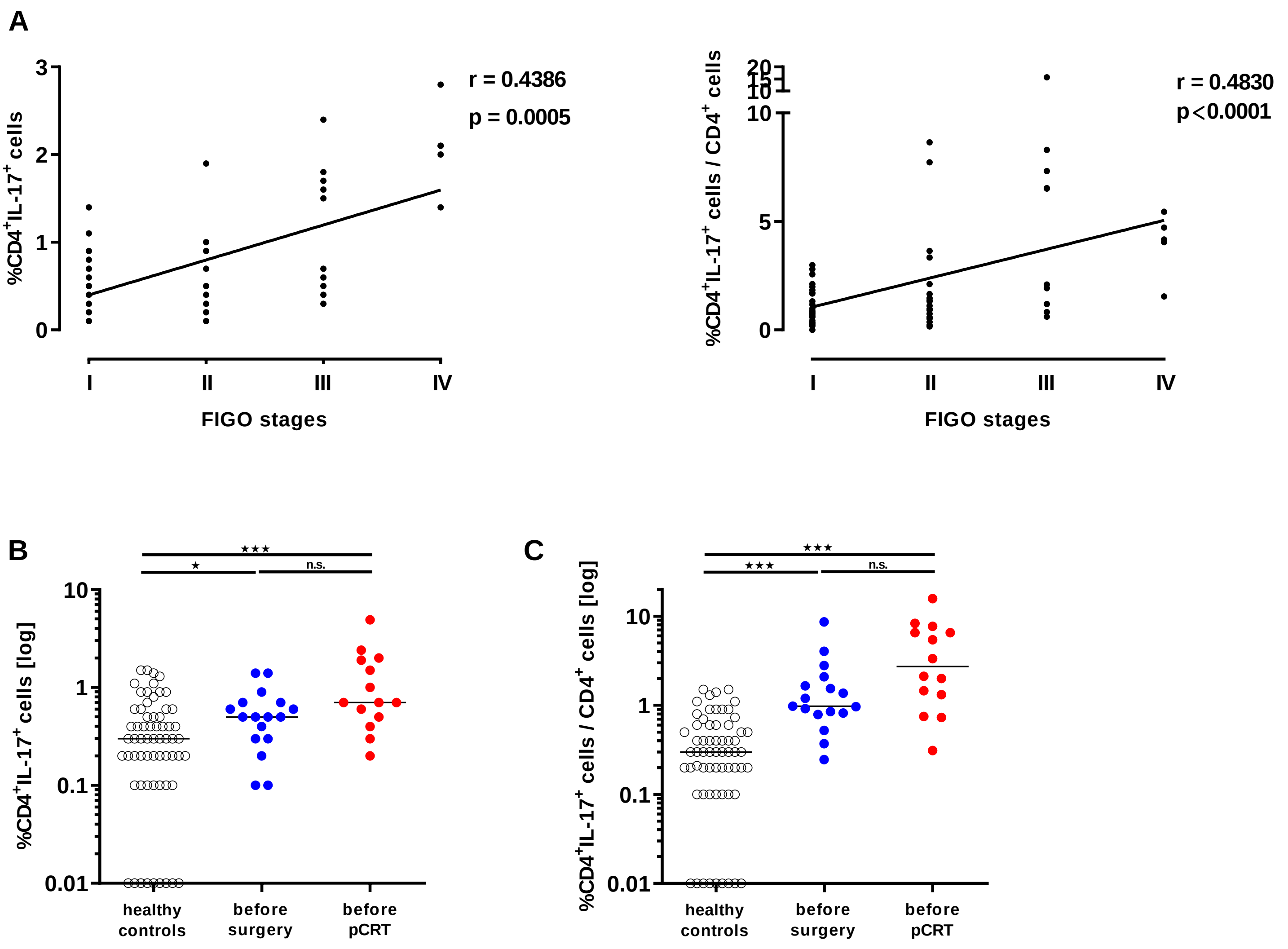


**Supplementary Figure S1: Pre-therapeutic frequencies of Th17 cells in the patients` blood correlated with tumor FIGO stages.** (A) Pre-therapeutic frequencies of Th17 cells or proportions of Th17 cells per CD4+ T cells of n=70 patients correlated with FIGO stages of the respective biopsies. (B) Patients recommended for adjuvant (blue dots) or primary CRT (red dots) exhibited significantly higher pre-therapeutic frequencies of Th17 cells or (C) proportions of Th17 cells per CD4+ T cells in comparison to healthy controls. Asterisks represent statistical significances: ★ $p \leq 0.05$; ★★★ $p \leq 0.001$.
