# Supplementary material for: Chemoradiotherapy‐induced increase in Th17 cell frequency in cervical cancer patients is associated with therapy resistance and early relapse
Source: Mol Oncol. 2021 Sep 13;15(12):3559–77. doi: 10.1002/1878-0261.13095 (PMC8637579; doi:10.1002/1878-0261.13095)
Supplement: Supplementary file 2 — Fig. S2. Cervical cancer cells exhibit different radiosensitivity. [file MOL2-15-3559-s002.pdf]

Supplementary Figure S2

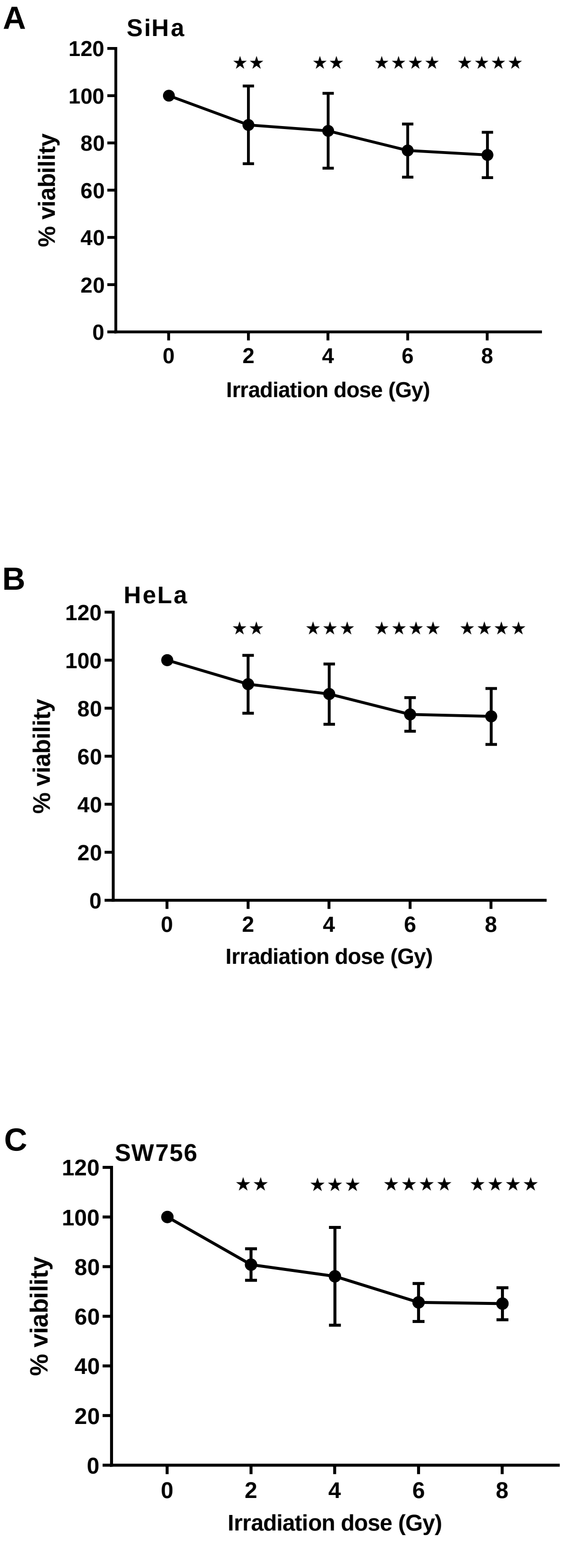

Supplementary Figure S2: Cervical cancer cells exhibit different radiosensitivity. SiHa (A), HeLa (B) and SW756 cells (C) were treated with increasing doses of irradiation (0-8 Gy). After 48 h cell viability was assessed by the neutral red uptake method. Shown are the results mean ± SD from three independent experiments performed in sextublicates. Asterisks represent statistical significances: ★★p ≤0.01; ★★★ p≤0.001; ★★★★ p≤0.0001.
