# Supplementary material for: Chemoradiotherapy‐induced increase in Th17 cell frequency in cervical cancer patients is associated with therapy resistance and early relapse
Source: Mol Oncol. 2021 Sep 13;15(12):3559–77. doi: 10.1002/1878-0261.13095 (PMC8637579; doi:10.1002/1878-0261.13095)
Supplement: Supplementary file 3 — Fig. S3. Impact of rhIL‐17 on the activation of STAT3 signaling and role of MAPK1 and MAPK14 on the IL‐17‐mediated resistance toward chemoradiotherapy in cervical cancer cells. [file MOL2-15-3559-s005.pdf]

Supplementary Figure S3

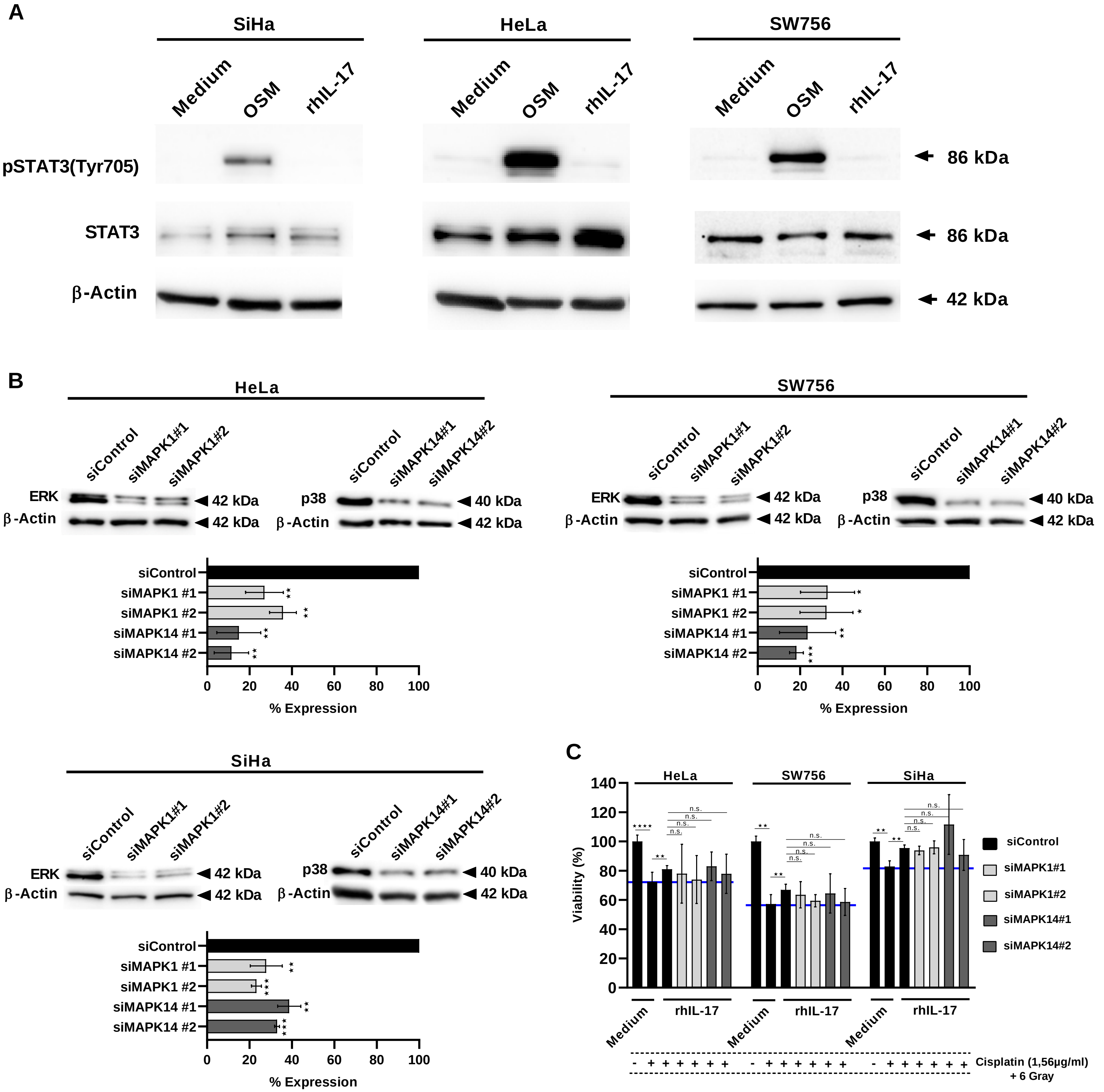

Supplementary Figure S3: Impact of rhIL-17 on the activation of STAT3 signaling and role of MAPK1 and MAPK14 on the IL-17-mediated resistance toward chemoradiotherapy in cervical cancer cells. (A) SiHa, HeLa and SW756 cells were stimulated with Oncostatin M (OSM), rhIL-17 or medium as a control for 15min und analyzed for pSTAT3(Tyr705) and STAT3 expression. β-Actin was used as a loading control. (B,C) SiHa, HeLa and SW756 cells were transfected with two specific siRNAs for MAPK1 (light grey bars) or MAPK14 (dark grey bars), respectively, or mock siRNA (black bars) as a control. (B) Whole cell extracts were analyzed for ERK and p38 expression in Western blot analysis. Equal loading was controlled using a β-actin-specific monoclonal antibody. Shown is one representative experiment out of n=3, bars represent quantification of n=3 independent experiments. Expression of mock siRNA transfected cells was set at 100%. (C) SiRNA transfected cells were stimulated with medium or rhIL-17. Cells were incubated with 1.56 µg/ml cisplatin for 2 h and irradiated with 6 Gy. After 48 h cell viability was assessed by the neutral red uptake method. Shown are the results mean ± SD from two independent experiments performed in triplicates. Blue lines mark levels of sensitivity toward chemoradiotherapy of the respective unstimulated cells. Asterisks represent statistical significances: n.s. not significant; ★★p ≤0.01; ★★★★ p≤0.0001.
