# Supplementary material for: Chemoradiotherapy‐induced increase in Th17 cell frequency in cervical cancer patients is associated with therapy resistance and early relapse
Source: Mol Oncol. 2021 Sep 13;15(12):3559–77. doi: 10.1002/1878-0261.13095 (PMC8637579; doi:10.1002/1878-0261.13095)
Supplement: Supplementary file 4 — Fig. S4. Analysis of AKT3 expression in different cervical cancer cells and specific knock down of AKT1 and AKT2. [file MOL2-15-3559-s010.pdf]

Supplementary Figure S4

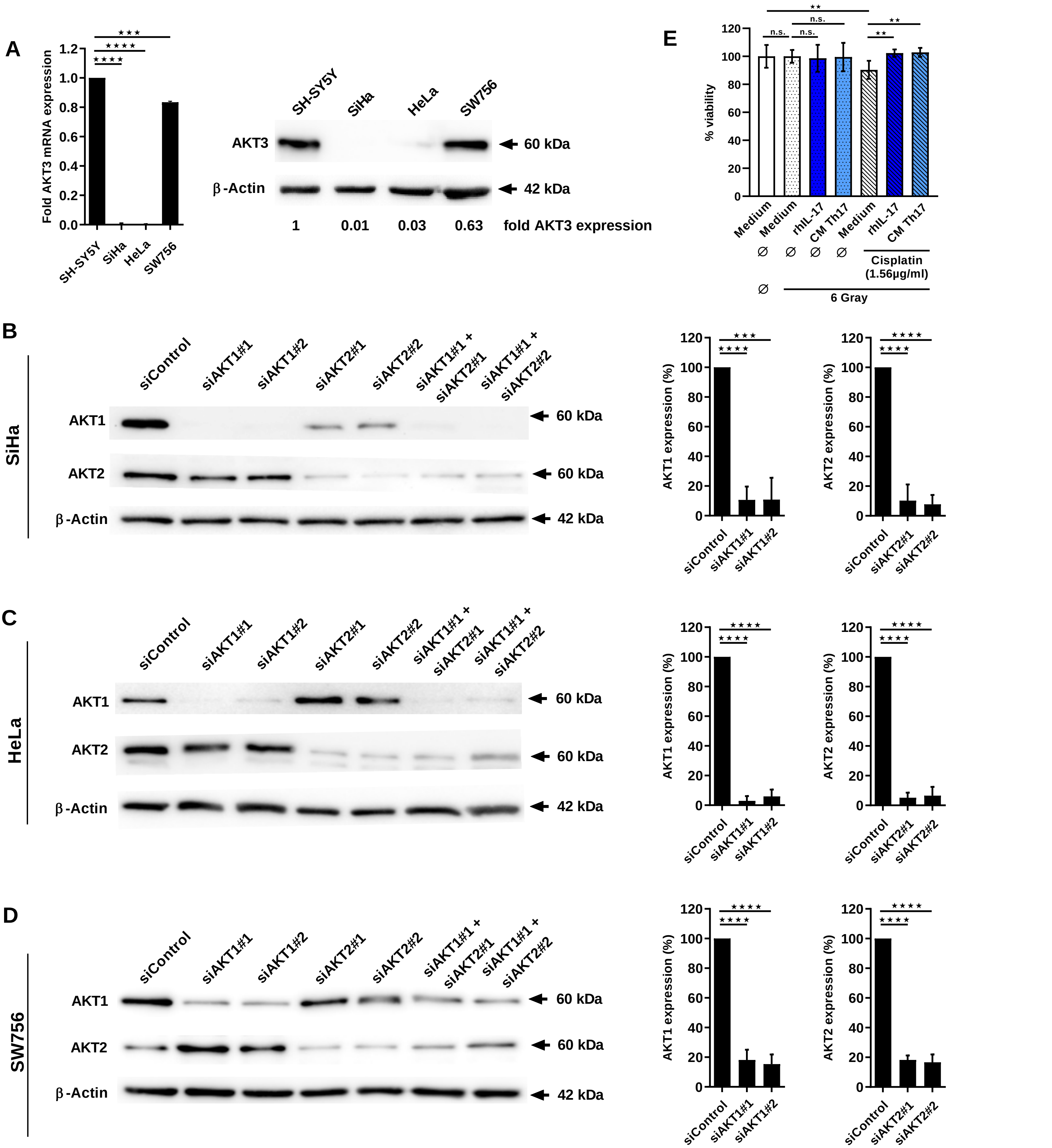

Supplementary Figure S4: Analysis of AKT3 expression in different cervical cancer cells and specific knock down of AKT1 and AKT2. (A) SH-SY5Y, SiHa, HeLa and SW756 cells were evaluated for AKT3 expression by qPCR (left) and western blot analysis (right) and normalized to RPL13A (left) or β-Actin expression (right). AKT3 expression of SH-SY5Y cells was set at 1. SiHa (B), HeLa (C) and SW756 cells (D) were transfected with two specific siRNAs for AKT1 or AKT2, respectively, both isoforms or mock siRNA as a control. Whole cell extracts were analyzed for AKT1 and AKT2 expression in Western blot analysis. Equal loading was controlled using a β-actin-specific monoclonal antibody. Shown is one representative experiment out of n=3, bars represent quantification of n=3 independent experiments. Expression of mock siRNA transfected cells was set at 100%. (E) CaSki were stimulated with medium (white bars), rhIL-17 (blue bars) or CM of *in vitro* generated Th17 cells (light blue bars) and irradiated with 6 Gy (dotted bars) or treated with 1.56 µg/ml cisplatin for 2 h and irradiated with 6 Gy (striped bars). After 48 h cell viability was assessed by the neutral red uptake method. Shown are the results mean ± SD from three independent experiments performed in triplicates. Asterisks represent statistical significances: ★★ p≤0.01; ★★★ p≤0.001; ★★★★ p≤0.0001.
