# Supplementary material for: Chemoradiotherapy‐induced increase in Th17 cell frequency in cervical cancer patients is associated with therapy resistance and early relapse
Source: Mol Oncol. 2021 Sep 13;15(12):3559–77. doi: 10.1002/1878-0261.13095 (PMC8637579; doi:10.1002/1878-0261.13095)
Supplement: Supplementary file 5 — Fig. S5. CD4+IL‐17+ cells infiltrate cervical SSCs and correlate with numbers of CD4+ and IL‐17+ cells, lymph node metastases and recurrent cervical cancers. [file MOL2-15-3559-s006.pdf]

Supplementary Figure S5

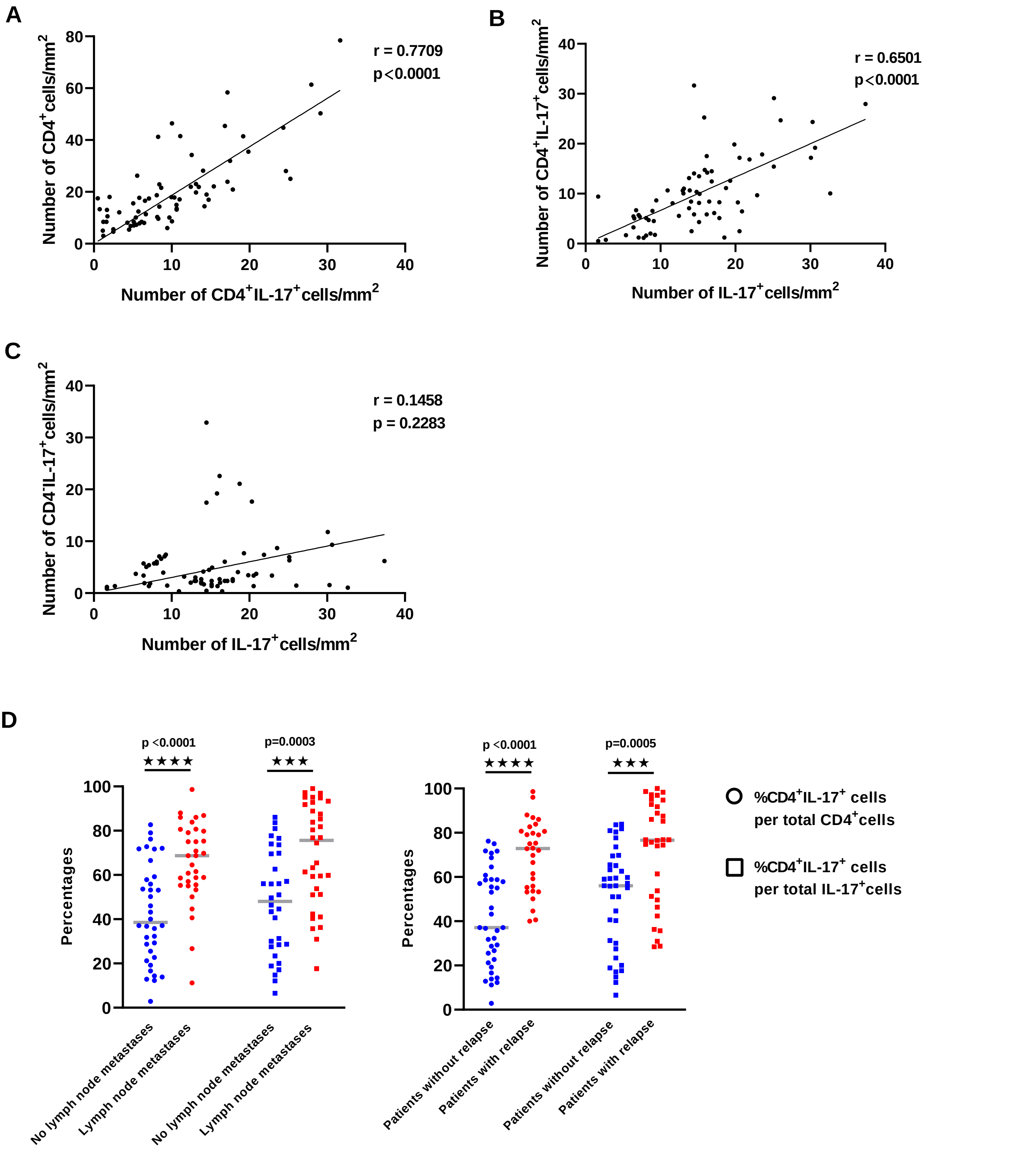

Supplementary Figure S5: CD4^{+}IL-17^{+} cells infiltrate cervical SSCs and correlate with numbers of CD4^{+} and IL-17^{+} cells, lymph node metastases and recurrent cervical cancers. Sections of human SCCs were costained for CD4 and IL-17 by immunofluorescence. (A-C) The number of CD4+ or IL-17+ cells was correlated with CD4+IL-17+ cells (A, B). (C) Numbers of CD4-IL-17+ cells were correlated with IL-17+ cells. (D) The percentages of CD4+IL-17+ cells per total CD4+ or IL-17+ cells correlated with lymph node metastasis or recurrent cervical cancers. Asterisks represent statistical significances: ★★★ p≤0.001; ★★★★ p≤0.0001.
