# Supplementary figures and images for: Chemoradiotherapy‐induced increase in Th17 cell frequency in cervical cancer patients is associated with therapy resistance and early relapse
Source: Mol Oncol. 2021 Sep 13;15(12):3559–77. doi: 10.1002/1878-0261.13095 (PMC8637579; doi:10.1002/1878-0261.13095)

# Supplementary Figure S6

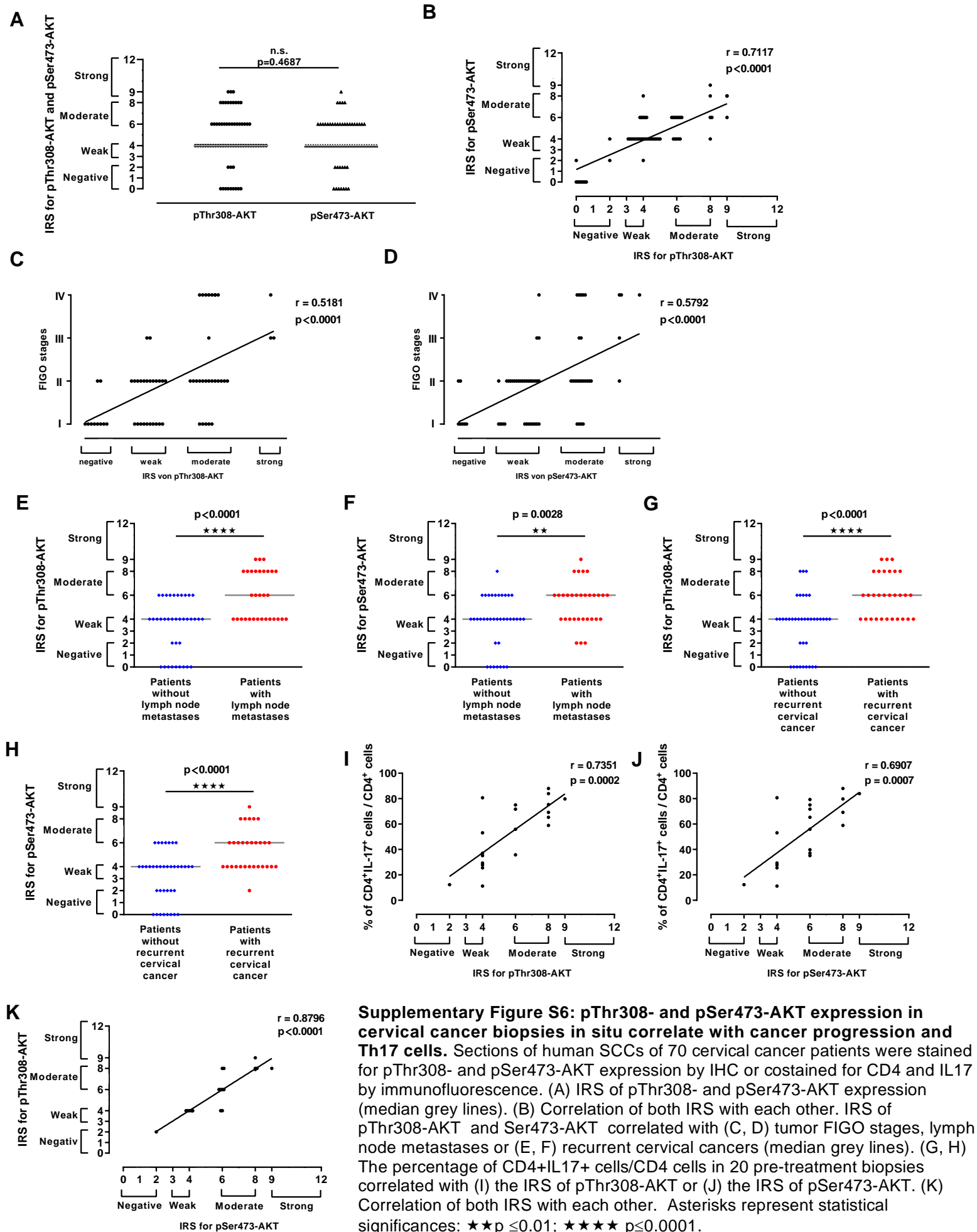

Supplement: Supplementary file 6 — Fig. S6. pThr308‐ and pSer473‐AKT expression in cervical cancer biopsies in situ correlate with cancer progression and Th17 cells. [file MOL2-15-3559-s008.pdf]
