# Supplementary material for: Chemoradiotherapy‐induced increase in Th17 cell frequency in cervical cancer patients is associated with therapy resistance and early relapse
Source: Mol Oncol. 2021 Sep 13;15(12):3559–77. doi: 10.1002/1878-0261.13095 (PMC8637579; doi:10.1002/1878-0261.13095)
Supplement: Supplementary file 7 — Fig. S7. FIGO stages as well as pre‐therapeutic Th17 frequencies did not significantly discriminate between patients with or without cervical cancer relapse. [file MOL2-15-3559-s007.pdf]

Supplementary Figure S7

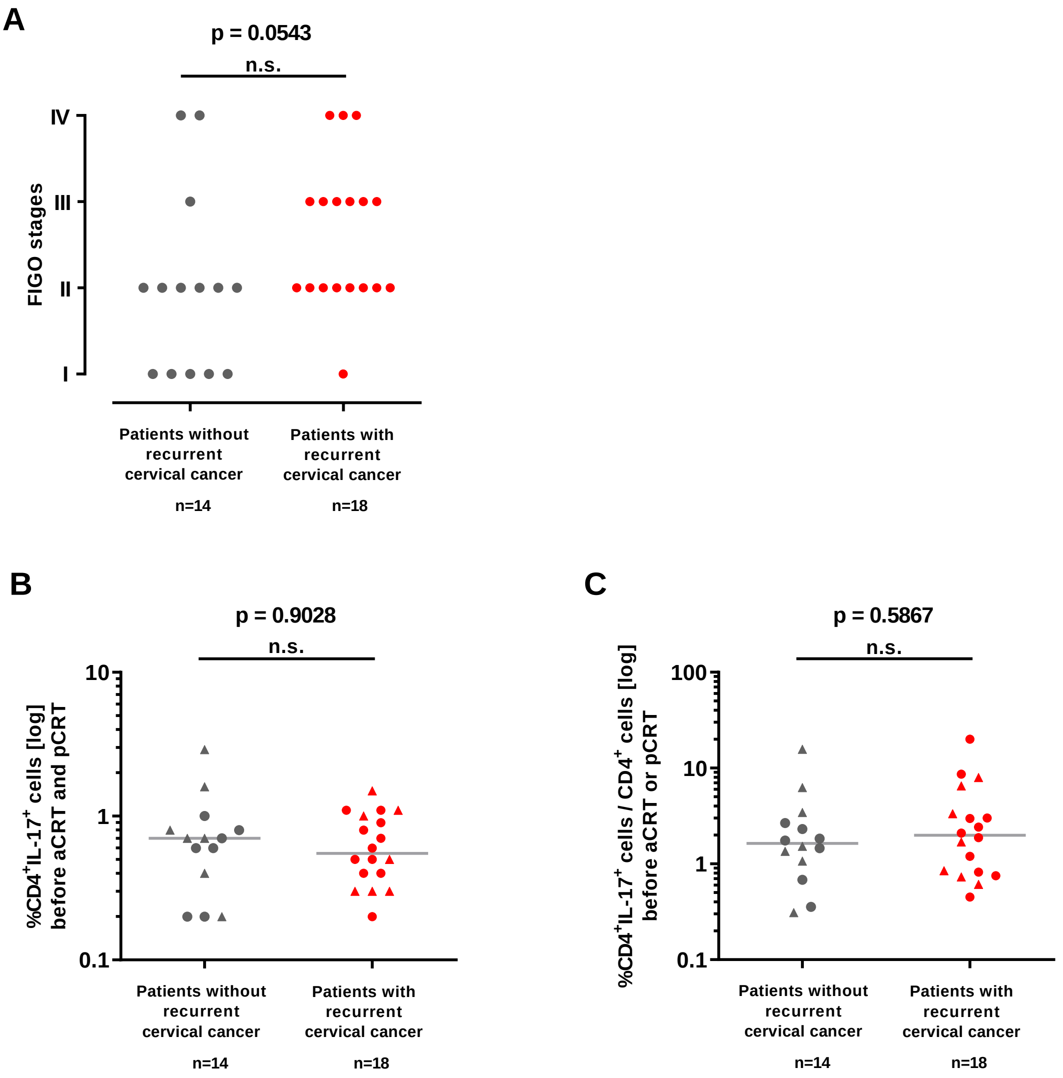

Supplementary Figure S7: FIGO stages as well as pre-therapeutic Th17 frequencies did not significantly discriminate between patients with or without cervical cancer relapse.
(A) FIGO stages of primary tumors, (B) frequencies of Th17 cells or (C) proportions of Th17 per CD4+ T cells before therapy were depicted for patients with or without recurrent cervical cancers.
