# Supplementary material for: Chemoradiotherapy‐induced increase in Th17 cell frequency in cervical cancer patients is associated with therapy resistance and early relapse
Source: Mol Oncol. 2021 Sep 13;15(12):3559–77. doi: 10.1002/1878-0261.13095 (PMC8637579; doi:10.1002/1878-0261.13095)
Supplement: Supplementary file 8 — Table S1. Pre‐ and post‐therapeutical stages according to the International Federation of Gynecology and Obstetrics (FIGO) or TNM categories. [file MOL2-15-3559-s004.pdf]

Supplementary Table S1: Pre- and post-therapeutical stages according to the International Federation of Gynecology and Obstetrics (FIGO) or TNM categories.

Patients	Pre-therapeutic FIGO stage	Primary chemoradiotherapy	Interval between pre-therapeutic staging and post-therapy classification	Post-therapeutic TNM classification	Response
1	IVB	Cisplatin + 50.4 Gy radiation	5 months	ypT4, N0	non
2	IV	Cisplatin + 50.4 Gy radiation	5 months	ypT4, N1	non
3	IV	Cisplatin + 59.4 Gy radiation	3 months	ypT4, N1	non
4	IVB	Cisplatin + 54 Gy radiation	3 months	ypT4, N1	non
5	IV	Cisplatin + 50.4 Gy radiation	4 months	ypT4, N1	non
6	IVA	Cisplatin + 50.4 Gy radiation	3 months	ypT2b, N1	partial
7	IV	Cisplatin + 45 Gy radiation	3 months	ypT1b1, N0	partial
8	IIB	Cisplatin + 45 Gy radiation	4 months	ypT1a2, No	partial
9	IIB	Cisplatin + 50.4 Gy radiation	3 months	ypT1b1, N0	partial
10	IVA	Cisplatin + 55.8 Gy radiation	4 months	ypT1b1, NX	partial
11	IIB	Cisplatin + 50.4 Gy radiation	3 months	ypT2a1, N1	partial
12	IIIB	Cisplatin + 50.4 Gy radiation	4 months	ypT2a, N0	partial
13	IIB	Cisplatin + 50.4 Gy radiation	4 months	ypT1b1	partial
14	IIIB	Cisplatin + 50.4 Gy radiation	4 months	ypT1b1	partial
15	III	Cisplatin + 50.4 Gy radiation	5 months	ypT0, N0	complete
16	IIB	Cisplatin + 50.4 Gy radiation	4 months	ypT0, No	complete
17	IV	Cisplatin + 50.4 Gy radiation	4 months	ypT0	complete
18	IIIB	Carboplatin + 50.4 Gy radiation	4 months	ypT0, N0	complete
19	IIB	Cisplatin + 50.4 Gy radiation	4 months	ypT0, No	complete
20	IIB	Cisplatin + 50.4 Gy radiation	4 months	ypT0	complete
