# Supplementary material for: Chemoradiotherapy‐induced increase in Th17 cell frequency in cervical cancer patients is associated with therapy resistance and early relapse
Source: Mol Oncol. 2021 Sep 13;15(12):3559–77. doi: 10.1002/1878-0261.13095 (PMC8637579; doi:10.1002/1878-0261.13095)
Supplement: Supplementary file 9 — Table S2. Immunoreactive Score (IRS) according to Remmele & Stegner. [file MOL2-15-3559-s001.pdf]

Supplementary Table S2: Immunoreactive Score (IRS) according to Remmele & Stegner.

A (% of positive cells)	B (Staining intensity)	A x B= IRS
0 = no positive cells	0 = no positive cells	0-2 = negative
1 = <10% of positive cells	1 = weak color reaction	3-4 = weak
2 = 10-50% of positive cells	2 = moderate color reaction	6-8 = moderate
3 = 51-80% of positive cells	3 = strong color reaction	9-12 = strong
4 = >80% positive cells		
