# Supplementary material for: Chemoradiotherapy‐induced increase in Th17 cell frequency in cervical cancer patients is associated with therapy resistance and early relapse
Source: Mol Oncol. 2021 Sep 13;15(12):3559–77. doi: 10.1002/1878-0261.13095 (PMC8637579; doi:10.1002/1878-0261.13095)
Supplement: Supplementary file 10 — Table S3. List of used materials. [file MOL2-15-3559-s009.pdf]

Supplementary Table S3: List of used materials.

ANTIBODIES			
**Antigen/Protein**	**Dilution/ concentration**	**Vendor**	**Identifier**
rabbit anti IL-17 polyclonal antibody	1:1000 for IF; Fig. 4	Abcam	#ab79056 RRID:AB_1603584
mouse anti CD4 monoclonal antibody, clone 4B12,	1:500 for IF; Fig. 4	Leica Biosystems,	#CD4-368-L-CE-H RRID:AB_563560
mouse anti human CD4 PE monoclonal antibody, clone RPA-T4	5µl pro test; Fig. 1	BD Biosciences	#555347 RRID:AB_395752
mouse anti human IL-17 APC monoclonal antibody, clone CZ8-23G1	2µl pro test; Fig. 1	Miltenyi Biotec,	#130-094-519 RRID:AB_10831356
Phospho-Akt (Thr308) (clone D25E6) XP® Rabbit mAb	1:1000 for WB; Fig. 3	Cell Signaling Technology	#13038 RRID:AB_2629447
Anti-AKT (phospho T308) antibody	1:250 for IHC; Fig.3	Abcam	#ab38449 RRID:AB_722678
Phospho-Akt (Ser473) (clone D9E) XP® Rabbit mAb	1:2000 for WB; Fig. 3<br>1:100 for IHC; Fig. 4	Cell Signaling Technology	#4060 RRID:AB_2315049
Akt (pan) (clone C67E7) Rabbit mAb	1:1000 for WB; Fig. 4	Cell Signaling Technology	#4691 RRID:AB_915783
rabbit anti Akt1 (clone C73H10)	1:1000 for WB; Suppl. Fig. S3	Cell Signaling Technology	#2938 RRID:AB_915788
rabbit anti Akt2 (clone D6G4)	1:1000 for WB; Suppl. Fig. S3	Cell Signaling Technology	#3063 RRID:AB_2225186
rabbit anti Akt3 (clone E1Z3W)	1:1000 for WB; Suppl. Fig. S3	Cell Signaling Technology	#14982 RRID:AB_2716311
mouse anti β-actin, clone AC-15	1:5000 for WB; Fig. 3 and Suppl. Fig. S2 and S3	Sigma-Aldrich,	#A5441 RRID:AB_476744
Human IL-17/IL-17A Antibody	1µg/ml for neutralization; Fig. 2	R&D Systems	#AF-317-SP RRID:AB_354463
normal goat IgG control	1µg/ml for neutralization; Fig. 2	R&D Systems	#AB-108-C RRID: AB_354267
Phospho-Stat3 (Tyr705) (D3A7) XP® Rabbit mAb	Supplementary Figure S2 1:2000 for WB	Cell Signaling Technology	#9145 RRID:AB_2491009
Stat3 (124H6) Mouse mAb	Supplementary Figure S2 1:1000 for WB	Cell Signaling Technology	#9139 RRID:AB_331757

CHEMICALS, PEPTIDES, AND RECOMBINANT PROTEINS		
Human IL-17, research grade	Miltenyi Biotec	#130-094-625
Human IL-6	PROSPEC	#CYT-213
Human TGF-β1, premium grade	Miltenyi Biotec	#130-095-067
Human IL-1β	PEPROTECH	#200-01B
Human IL-23, research grade	Miltenyi Biotec	#130-095-757
RPMI-1640 medium	Sigma-Aldrich	#R8758
Dulbecco's Modified Eagle's Medium - high glucose	Sigma-Aldrich	#D5796
Dulbecco's Phosphate Buffered Saline	Sigma-Aldrich	#D8537
Fetal Bovine Serum	Sigma-Aldrich	#F7524
Fetal Bovine Serum, qualified	Gibco	#10270-106
Sodium pyruvate solution	Sigma-Aldrich	#S8636
Amersham™ Protran® Western blotting membranes, nitrocellulose	GE Healthcare Life Science	#10600002
Neutral Red	Sigma-Aldrich	#N4638
QIAzol Lysis Reagent	QIAGEN	#79306
Opti-MEM™, GlutaMAX™ I	Thermo Fisher	#51985034
Lipofectamine™ RNAiMAX	Thermo Fisher	#13778150
Pancoll human	PAN biotech	#P04-601000
Phorbol 12-myristate 13-acetate (PMA)	Sigma-Aldrich	#P8139
Ionomycin	Sigma-Aldrich	#I0634
Brefeldin A	Sigma-Aldrich	#B6542
Paraformaldehyde	Sigma-Aldrich	#P6148

COMMERCIAL ASSAYS

BD Cytofix/Cytoperm™	BD Bioscience	#554714
Maxima Reverse Transcriptase	ThermoFisher Scientific	#EP0742
Fast Start Taq DNA Polymerase dNTPack	Roche	#4738357001
ImmPRESS® HRP Horse Anti-Rabbit IgG Polymer Detection Kit, Peroxidase	Vector Laboratories	#MP-7401
Invitrogen™ Molecular Probes™ TSA™ Kit 2, with HRP-Goat Anti-Mouse IgG and Alexa Fluor™ 488 Tyramide	Life Technology	#T20912
Invitrogen™ Molecular Probes™ TSA™ Kit 13, with HRP-Goat Anti-Rabbit IgG and Alexa Fluor™ 546 Tyramide	Life Technology	#T20923
Naive CD4$^+$ T Cell Isolation Kit II , human	Miltenyi Biotec	#130-094-131
T Cell Activation/Expansion Kit, human	Miltenyi Biotec	#130-091-441
IL-17 Secretion Assay – Cell Enrichment and Detection Kit (PE), human	Miltenyi Biotec	#130-094-542
SuperSignal West Dura Substrate	Thermo Fisher	#34076
Human IL-17 DuoSet ELISA	R&D Systems	#DY317
DuoSet ELISA Ancillary Reagent Kit 2	R&D Systems	#DY008
Mycotrace Pcr Detection Kit	PAA	#Q050-020
Venor®GeM Classic	Minerva Biolabs	#11-1025
**EXPERIMENTAL MODELS: CELL LINES**		
Human HPV-16 positive cervical cancer cell line SiHa	ATCC	CVCL_0032
Human HPV-16 positive cervical cancer cell line CaSki	ATCC	CVCL_1100
Human HPV-18 positive cervical cancer cell line HeLa	ATCC	CVCL_0030
Human HPV-18 positive cervical cancer cell line SW756	ATCC	CVCL_1727
SH-SY5Y	ATCC	CRL-2266
**OLIGONUCLEOTIDES**		
ON-TARGETplus siRNA AKT1 #12, targeting sequence: CAAGGGCACUUUCGGCAAG	Horizon Discovery	J-003000-12
ON-TARGETplus siRNA AKT1 #13, targeting sequence: UCACAGCCCUGAAGUACUC	Horizon Discovery	J-003000-13
ON-TARGETplus siRNA AKT2 #09, targeting sequence: ACACAAGGUACUUCGAUGA	Horizon Discovery	J-003001-09
ON-TARGETplus siRNA AKT2 #10, targeting sequence: GCAAGGCACGGGCUAAAGU	Horizon Discovery	J-003001-10
ON-TARGETplus Non-targeting siRNA #2; targeting sequence: UGGUUUACAUGUUGUGUGA	Horizon Discovery	D-001810-02
ON-TARGETplus siRNA MAPK1 #11, targeting sequence: UCGAGUAGCUAUCAAGAAA	Horizon Discovery	J-003555-11
ON-TARGETplus siRNA MAPK1 #14, targeting sequence: ACACCAACCUCUCGUACAU	Horizon Discovery	J-003555-14
ON-TARGETplus siRNA MAPK14 #20, targeting sequence: GGAAUUCAAUGAUGUGUAU	Horizon Discovery	J-003512-20
ON-TARGETplus siRNA MAPK14 #21, targeting sequence: UCUCCGAGGUCUAAAGUAU	Horizon Discovery	J-003512-21
qRTPCR primer for human specific Akt3<br>Forward 5`-3`: TTGCTTTCAGGGCTCTTGAT<br>Reverse 5`-3`: CATAATTTCTTTTGCATCATCTGG	This study	Sigma Aldrich
qRTPCR primer for RPL13A<br>Forward 5`-3`: AGCGGATGAACACCAACC<br>Reverse 5`-3`: TTTGTGGGGCAGCATACTC	This study	Sigma Aldrich
**SOFTWARE**		
Image Lab	Bio Rad	https://www.bio-rad.com/de-de/product/image-lab-software
VIS (Visiopharm Integrator sytem)	Visiopharm	https://visiopharm.com/
cellSens Dimension	Olympus	https://www.olympus-lifescience.com/de/software/cellsens/
GraphPad Prism8	GraphPad	https://www.graphpad.com/scientific-software/prism/

BD FACSDiva™ Software	BD Bioscience	https://www.bdbiosciences.com/en-us/instruments/research-instruments/research-software/flow-cytometry-acquisition/facsdiva-software
